# Investigating the Mechanism of Germinal Center Shutdown

**DOI:** 10.3389/fimmu.2022.922318

**Published:** 2022-07-14

**Authors:** Theinmozhi Arulraj, Sebastian C. Binder, Michael Meyer-Hermann

**Affiliations:** ^1^ Department of Systems Immunology, Braunschweig Integrated Centre of Systems Biology, Helmholtz Centre for Infection Research, Braunschweig, Germany; ^2^ Institute for Biochemistry, Biotechnology and Bioinformatics, Technische Universität Braunschweig, Braunschweig, Germany

**Keywords:** germinal center shutdown, chronic germinal centers, vaccination, antibody responses, mathematical modeling

## Abstract

Germinal centers (GCs) are transient structures where affinity maturation of B cells gives rise to high affinity plasma and memory cells. The mechanism of GC shutdown is unclear, despite being an important phenomenon maintaining immune homeostasis. In this study, we used a mathematical model to identify mechanisms that can independently promote contraction of GCs leading to shutdown. We show that GC shutdown can be promoted by antigen consumption by B cells, antigen masking by soluble antibodies, alterations in follicular dendritic cell (FDC) network area, modulation of immune complex cycling rate constants, alterations in T follicular helper signaling, increased terminal differentiation and reduced B cell division capacity. Proposed mechanisms promoted GC contraction by ultimately decreasing the number of B cell divisions and recycling cells. Based on the in-silico predictions, we suggest a combination of experiments that can be potentially employed by future studies to unravel the mechanistic basis of GC shutdown such as measurements of the density of pMHC presentation of B cells, FDC network size per B cell, fraction of cells expressing differentiation markers. We also show that the identified mechanisms differentially affect the efficiency of GC reaction estimated based on the quantity and quality of resulting antibodies.

## Introduction

Germinal centers (GCs) are highly organized structures in secondary lymphoid organs that support affinity maturation of B cells by iterative rounds of mutation and selection (1). A mature GC is composed of two compartments – a Dark zone (DZ) and a Light zone (LZ) (2, 3). In the DZ, dividing B cells called centroblasts mutate their B cell receptor (BCR) gene by a process termed somatic hypermutation. B cells are referred as centrocytes in the LZ, where the B cells are selected based on the BCR affinities. For this purpose, LZ comprises a network of follicular dendritic cells (FDCs) displaying immune complexes (ICs) and T follicular helper (Tfh) cells. Acquisition of ICs from FDCs and peptide-MHC (pMHC) presentation to Tfh cells, prevents apoptosis of centrocytes (4) and promotes recycling to the DZ for cell divisions (5) or induces differentiation into precursors of memory or plasma cells that leave the GCs (6, 7). Mechanisms involved in selection and cell fate decisions of GC B cells are actively being elucidated (6, 8–11).

This evolutionary process of B cells lasts only for a limited period of time as GCs are transient and undergo shutdown. The lifetime of single GCs in a lymphoid organ is unknown, but collective GC responses in a lymphoid organ lasts for a period of 3 weeks in the case of model protein antigens and more than a month in chronic viral infections (12–14). Therefore, the exact lifetime of GC responses varies depending on the immunization conditions such as the antigen and adjuvant used (15–17). In a normal infection or immunization, shutdown of GCs affects the quality and quantity of antibody responses by limiting the timeline of affinity maturation and generation of output cells. Consequently, shutdown of GCs is an attractive target for the therapeutic modulation of GC responses. However, GC shutdown remains poorly understood and the underlying mechanism is unresolved since decades, thus, hindering the identification of strategies to efficiently modulate GC lifetime.

GC contraction occurs due to a shift in the balance between proliferation, apoptosis and terminal differentiation of B cells when apoptosis and differentiation overtake the proliferation (18). These alternative B cell fates are modulated by interactions with FDCs and Tfh cells (19). Proliferation of GC B cells is controlled by the transcription factor c-Myc, deficiency of which can cause premature GC shutdown (20) suggesting the importance of continued cell divisions in GC maintenance. Similarly, the absence of FDCs or Tfh cells lead to the dissolution of established GC reactions by inducing GC B cell apoptosis (21–23). Dysregulation of B cell proliferation, apoptosis and terminal differentiation are commonly observed in GC-derived B cell lymphomas (24–27).

These experimental observations suggest the role of different cell types and B cell intrinsic factors in controlling GC maintenance but the exact mechanism behind natural GC shutdown is unknown (28). Nevertheless, recent studies have identified progressive changes occurring during the GC reaction, in FDCs, Tfh cells, T follicular regulatory (Tfr) cells and GC B cells. FDCs undergo changes in morphology and surface marker expression (2, 29, 30). Tfh cells are selected depending on their ability to recognize pMHC presented by B cells (31) and undergo changes in the expression of CD40L and cytokines (32). Dynamics of Tfr cells peak at late stages of GC reaction (33, 34), highlighting the potential role in GC shutdown. Repeated interactions of these cell types might lead to alterations in the GC B cell fate and cause GC shutdown. But the mutual dependence of different cell types and the mechanisms underlying the characteristic changes during GC evolution are only partly understood. It is also unclear whether these dynamic changes are individually sufficient to terminate GCs.

Poor understanding of GC shutdown is partly due to technical challenges and complex behavior of GCs. Mathematical modeling was an integral part of GC research and contributed to our understanding of the mechanisms behind GC B cell selection (11, 35), predicted the existence of recycling (36, 37), predicted the role of soluble antibodies in GC shutdown (38), used to devise strategies for induction of broadly neutralizing antibodies (39, 40) and for enhancing vaccination responses (41, 42). Based on an ordinary differential equation model of GC reaction, antigen limitation was proposed as a mechanism of GC shutdown over limiting T cell help (43). Previously, mathematical models were also used to establish rules governing the differentiation of centroblasts to centrocytes, selection of centrocytes and recycling of selected centrocytes by comparing the consistency with experimental GC kinetics and ability to recapitulate GC termination (44). Considering the new findings and recent advances in the understanding of GC evolution, there is a potential to better characterize the mechanism of GC shutdown. In this study, we consider evidence from various experimental studies and identify mechanisms that can individually drive GC shutdown. We suggest a number of experiments to test the proposed mechanisms. Finally, we discuss the implications of GC shutdown on the quality and quantity of GC output in different mechanisms.

## Methods

### Overview of the GC Model

Mechanisms of GC shutdown were implemented in the framework of GC model *hyphasma* (45–47). The base model includes a three-dimensional discrete lattice with CXCL12 and CXCL13 chemokine gradients. An agent-based approach was used and individual B cells, Tfh cells and FDCs were randomly placed in the lattice and simulated. Both the number of FDCs and Tfh cells were assumed to be 200. FDCs comprise a soma and six-dendrites of length 40 µm that extend from the soma of each FDC and therefore, occupy multiple lattice sites. Position of soma of the FDCs were randomly chosen in the LZ region of the lattice. Each FDC was loaded with 3000 antigen portions and the antigen amount was equally distributed among the lattice sites occupied by the FDC.

A four-dimensional shape space (48) was used for BCR affinity representation. Mutations were modelled by a random shift in the shape space position to neighboring sites. GC B cells undergo state transition corresponding to different stages. B cells were randomly incorporated in the lattice with an influx rate of 2 cells per hour for the first 4 days. Seeder B cells belong to the state *DZ B cell*, where each cell undergoes six divisions each and mutate with a probability 0.5 (49, 50). *DZ B cell* switches to the *Unselected* state after divisions and go through an antigen collection phase for 0.7 hours. Antigen acquisition by B cell reduces the antigen amount on FDCs. Multiple antigen collection events were allowed while constraining the time interval between subsequent antigen collection events to 0.02 hours. *Unselected* to *FDCselected* transition signifies successful antigen uptake and *FDCselected* cells establish contacts with Tfh cells.

Each interaction of B cell with Tfh lasts for 6 minutes. Acquisition of Tfh signals occurs when the Tfh polarizes to the B cell. When multiple B cells are bound to a given Tfh cell, the Tfh cell is assumed to polarize only towards the B cell with highest antigen uptake. Signals acquired from Tfh is integrated in a given B cell. If the signals acquired reach a threshold of 0.5 h at the end of 3 hours, then the B cell is *Selected*. *Selected* cells acquire *DZ B cell* state and divide again. Seventy-two percent of the divisions were assumed to be asymmetric in terms of antigen distribution (45, 51). It is assumed that one of the daughter cells retains antigen and differentiates into an output cell and the other cells switch to *Unselected* state and continue antigen collection.

### Alternate Assumptions Considered

Different assumptions were considered to test the validity of the predictions by varying the determinants of Tfh signaling intensity and number of cell divisions.

#### Number of Divisions

Number of divisions of recycling GC B cells *n_div_* was assumed to depend either on the antigen uptake *p* or the Tfh signals collected *T_sig_* according to the following equations.


Equation 1 $$n_{\text{div}}\left(p\right)=n_{\text{min}}+\left(n_{\text{max}}-n_{\text{min}}\right)\frac{p^{\text{n}}}{p^{\text{n}}+K^{\text{n}}}$$

Equation 2 $$n_{\text{div}}\left(T_{\text{sig}}\right)=n_{\text{min}}+\left(n_{\text{max}}-n_{\text{min}}\right)\frac{T_{{\text{sig}}^{n}}}{T_{{\text{sig}}^{n}}+K^{\text{n}}}$$

*K* is the amount of antigen captured or Tfh signals acquired when $n_{\text{div}}=\frac{\;\;n_{\text{min}}+n_{\text{max}}}{2}$ . We used a Hill coefficient n = 2, a maximum number of divisions *n*_max_ = 6 and a minimum number of divisions *n*_min_ = 1.

#### Tfh Signaling Intensity

The Tfh signaling intensity was assumed to depend directly or only indirectly on the pMHC presentation of GC B cells. In the first scenario, upon successful polarization, Tfh signals *T*_sig_ = *T*_max_ *J(p)* delivered to the B cell is a function of pMHC presentation *p* and calculated as


Equation 3 $$J=J_{\text{min}}+\left(J_{\text{max}}-J_{\text{min}}\right)\frac{p^{\text{n}}}{p^{\text{n}}+K_{{\text{p}}^{n}}}$$

with *J*_min_ = 0, *J*_max_=3, n = 2 and *K*_p_ =7.1.

For the indirect dependence, Tfh signals delivered to the B cell is simply a measure of the time period during which the Tfh cell is polarized to the B cell (*J* = 1). For direct and indirect dependency, the Tfh cell polarizes towards the B cell that has highest pMHC presentation in the presence of multiple interacting B cells leading to an indirect dependence of Tfh signals on antigen collected under conditions of high B cell competition for Tfh help.

### Implementation of Different Mechanisms

A control simulation was considered to test the ability of different mechanisms to shutdown GCs. In the control, no mechanism of GC shutdown is explicitly considered and decrease in free antigen due to B cell consumption is neglected. Therefore, the GC reaction does not terminate.

#### Mechanism 1: Antigen Consumption

Acquisition of antigen by B cells decreases the amount of antigen in the FDCs. This gradual decrease in antigen is assumed to be the cause of GC shutdown. To vary the speed of GC contraction, the amount of antigen portions acquired by GC B cell upon a single encounter with a FDC is varied relative to the total antigen amount. In the control simulation corresponding to this mechanism, the amount of antigen consumed by GC B cell was assumed to be extremely low relative to the total amount of antigen and therefore, the decrease in antigen amount due to B cell consumption was negligible.

#### Mechanism 2: Antibody Feedback

Output cells exiting the GC area were assumed to differentiate into plasma cells with a rate constant corresponding to a half-life of 24 h. Plasma cells secrete antibodies at a rate of *k*_1_ = 3 x 10^-18^ mol per hour per cell (52). Antibodies were distributed in 11 bins, that reflect differences in the affinities of antibodies produced. Each bin (*i*) corresponds to a different *k*_off_ and same *k*_on_ thus leading to different affinities such that the dissociation constant is in the range of 10^-5.5^ to 10^-9.5^ M. Antibodies were assumed to undergo degradation with a rate constant of $k_{2}=\frac{\ln2}{14\;}\;{\text{day}}^{-1}$ . The antibody concentration in each bin *A*(*i*) is diluted over a volume *V* of 10 ml, as it is assumed to distribute throughout the circulatory system and is calculated as follows.


Equation 4 $$\frac{dA\left(i\right)}{dt}=\frac{k_{1}}{V}n_{\text{p}}\left(i\right)-k_{2}A\left(i\right)$$

In this equation, *n*_p_(*i*) is the number of plasma cells with affinity corresponding to bin *i*.

Masking of antigen by antibodies in Equation 4 leads to a decrease in the free antigen concentration *G*_FDC_, while dissociation of antibody increases the concentration of free antigen. Changes in the concentration of soluble antibodies by the formation of ICs is neglected. The rate of change of masked antigen concentration *C*_FDC_ follows


Equation 5 $$\frac{dC_{\text{FDC}}\left(i\right)}{dt}=k_{\text{on}}G_{\text{FDC}}NA\left(i\right)-k_{\text{off}}\left(i\right)C_{\text{FDC}}\left(i\right)$$

To vary the strength of antibody feedback, a scaling factor (*N*) is used to modulate the concentration of antibodies promoting feedback in the above equation. This scaling factor can be interpreted as the number of synchronously initialized GCs (53, 54). In the control simulation, masking of antigen by soluble antibodies is not considered.

#### Mechanism 3: FDC Contraction

Length of FDC dendrites was assumed to increase at early time points and decrease thereafter. At the start of the simulation, the length of FDC dendrites were assumed to be 5 µm. Their length was increased at a constant rate of 0.166 µm per hour until day 7 after GC onset. After day 7, the FDC dendrites were shortened at a constant rate. Different values for the rate of FDC dendrite contraction *k*_c_ were considered. It is assumed that the extension and contraction of FDCs do not affect the total antigen concentration on the surface of FDCs. After each FDC extension or contraction event, the total amount of antigen in the FDC’s previous state was redistributed on all the FDC sites in the current state. In the control simulation, growth of FDC dendrites was considered as stated above but the FDC contraction rate was assumed to be 0.

#### Mechanism 4: Ag Internalization

Cycling of ICs in FDCs was considered in this mechanism as implemented in (55). Antigen on FDCs is assumed to be present in either of the two states – Interior or Surface. Surface antigen *A*_surface_ is assumed to be available for GC B cell uptake. We assumed that the rest of the antigen is in an internalized state *A*_total_-*A*_surface_. The cycling rate constants *k*_int_ =1/21 min^-1^ and *k*_ext_ =1/36 min^-1^ were estimated from PE-IC data of (56) in (55) and are used at the start of the simulation. The amount of surface antigen on the FDCs due to internalization and externalization varies according to


Equation 6 $$\frac{dA_{\text{surface}}}{dt}=k_{\text{ext}}\left(A_{\text{total}}-A_{\text{surface}}\right)-k_{\text{int}}A_{\text{surface}}$$

As the GC progresses, *k*_ext_ is assumed to be modulated in a time *t* dependent manner according to


Equation 7 $$k_{\text{ext}}\left(t\right)=k_{\text{ext}}\left(0\right)\left(1-\frac{t^{\text{n}}}{t^{\text{n}}+K_{{\text{ext}}^{n}}}\right)$$

with the Hill coefficient n= 2. *k*_ext_ , the time point where *k*_ext_ (*t*) = *k*_ext_ (0)/2, was varied (=100, 150, 200 h for A1; 150, 200, 250 h for A2; 60, 80, 100 h for A3; 200, 250, 300 h for A4) in the simulations. In the control simulation, *k*_int_ and *K*_ext_ remain constant throughout the GC reaction.

#### Mechanism 5: Tfh Signaling Capacity

The intensity of the maximum Tfh signaling *T*_max_ is assumed to decrease according to the following Hill function in a time dependent manner *t*.


Equation 8 $$T_{\text{max}}\left(t\right)=T_{\text{max}}\left(0\right)\left(1-\frac{t^{\text{n}}}{t^{\text{n}}+K_{{\text{T}}^{n}}}\right)$$

with *n*=2. *K*_T_, the time point where *T*_max_(*t*) = *T*_max_(0)/2 , was varied (= 400, 600, 800 h) to adjust the speed of GC contraction. In the control simulation, the Tfh signaling intensity is assumed to be constant throughout the simulation.

#### Mechanism 6: Terminal Differentiation

Tfh selected cells were assumed to differentiate to output cells and exit the GC with probability *F*, without recycling to the DZ for cell cycle re-entry. Different possibilities were considered for this mechanism, where the terminal differentiation in the LZ is antigen uptake (*p*), Tfh signal (*T*_sig_) or time dependent (*t*). In antigen uptake and Tfh dependent terminal differentiation, the probability of differentiation was calculated according to


Equation 9 $$F=\frac{X^{\text{n}}}{X^{\text{n}}+K_{F}^{\text{n}}}$$

where *X*=*p* and *T*_sig_, respectively, and n=2. The value of *K*_F_ (= 3, 5, 9 antigen units in antigen dependent differentiation and 0.5, 1, 1.5 Tfh signal units in Tfh dependent differentiation) was varied in the simulations. In time dependent (*t*) terminal differentiation, the probability of terminal differentiation follows


Equation 10 $$F=1-e^{-kt}$$

where *k* (=0, 0.002, 0.003, 0.004 h^-1^) was varied to adjust the speed of GC shutdown. In the control simulations, *F*=0 and therefore, 100 % of selected cells recycle back to the DZ for cell cycle re-entry.

#### Mechanism 7: B Cell Division Capacity

The number of divisions of GC B cells was assumed to depend on the number of DZ-LZ cycles underwent, in addition to the dependence on antigen uptake and Tfh signals acquired.

The value of *K* in Equations 1 and 2 was assumed to be a function of the number of DZ-LZ cycles *N*_cyc_ and calculated as


Equation 11 $$K=K_{\text{min}}+\left(K_{\text{max}}-K_{\text{min}}\right)\frac{N_{\text{cyc}}^{\text{n}}}{N_{\text{cyc}}^{\text{n}}+K_{{\text{k}}^{n}}}$$

The value of *K* is minimum *K*_min_ at the start of the simulation. In the control simulation, the value of *K* remains constant and equal to *K*_min_ throughout the simulation. We used n=2, *K*_min =_ 1, 1, 0.5, 0 in A1-A4, respectively, and *K*_max_ = 25, 25, 4.5, 6 in A1-A4, respectively. The value of *K*_K_ (= 2, 5, 8 for A1-A3; 7, 5, 3 for A4) was varied in the simulations.

### Calculation and Normalization of Readouts

The average number of divisions of selected or recycled B cells were calculated at one-hour time windows during the course of the reaction.

To calculate Immune Power (IP) or efficiency of GC reactions (53, 54), a test antigen concentration *R* =10^-5^ M was considered. The fraction of test antigen bound *R*_bound_ by soluble antibodies *A*(*i*) produced from the GCs was calculated using the following steady state approximation which assumes that the concentration of test antigen is much higher than the concentration of antibodies:


Equation 12 $$R_{\text{bound}}\left(i\right)=\frac{A\left(i\right)R}{K\left(i\right)+R}$$

Equation 13 $$IP=\frac{\sum R_{\text{bound}}\left(i\right)}{R}$$

## Results

### Mechanisms of GC Shutdown

**Figure 1:**
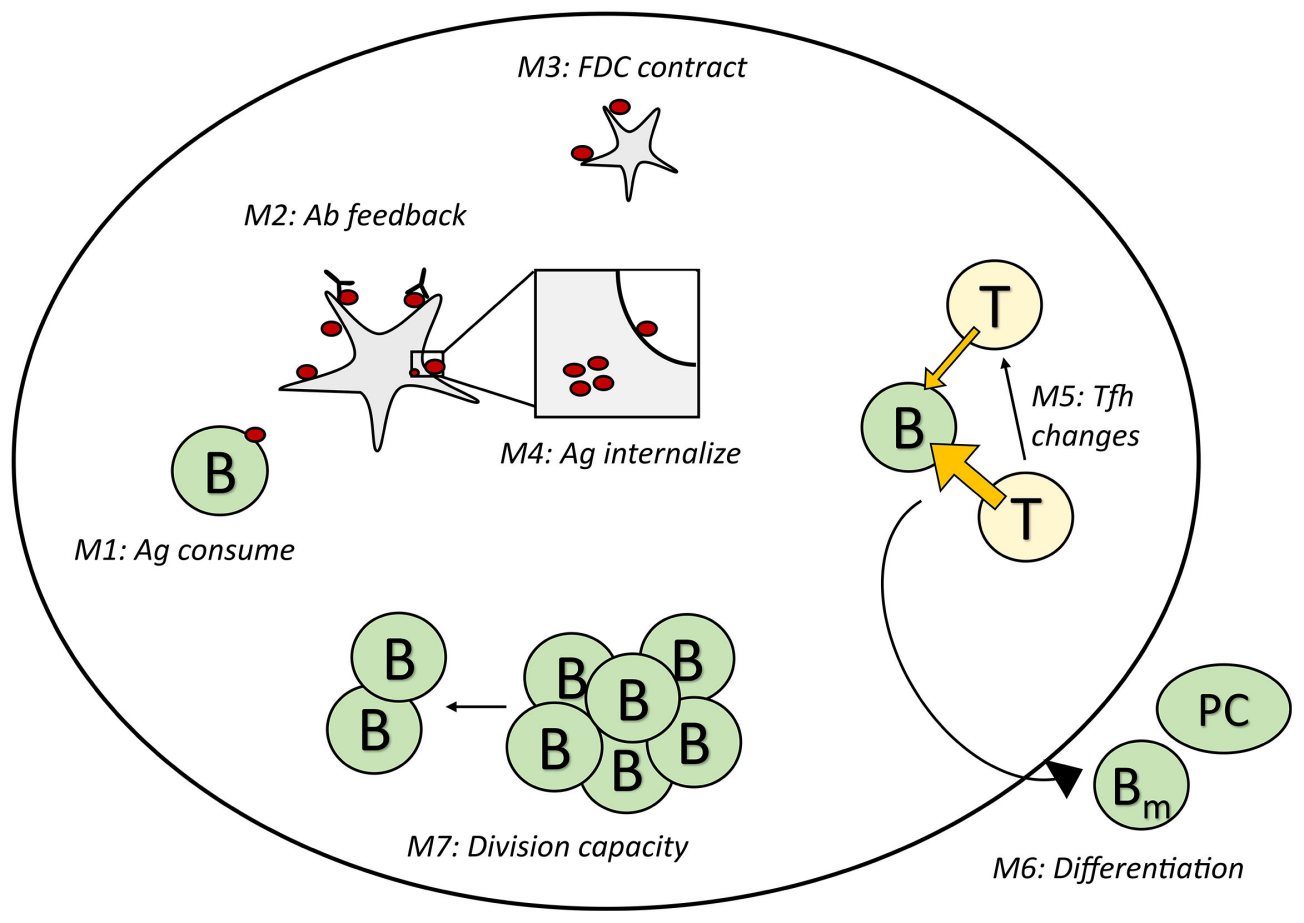
Schematic representation of the GC shutdown mechanisms. In mechanisms M1-M4, antigen limitation arises due to the consumption of antigen by B cells, masking of antigen by soluble antibodies (antibody feedback), contraction of FDC network and changes in antigen cycling rate constants leading to increased internalization of antigen, respectively. In M5, Tfh signaling intensity decreases leading to limiting T cell help. In M6, increased terminal differentiation of GC B cells into memory/plasma cells leads to increased exit from GCs. In M7, B cells have limited capacity to divide leading to decreased proliferation over time. GC, Germinal center; FDC, Follicular Dendritic Cell; T/Tfh, T follicular helper cell; Ab, Antibody; Ag, Antigen; PC, Plasma cell; B_m_, Memory B cell; B, Germinal center B cell.

We performed *in-silico* simulations by implementing seven different mechanisms of GC shutdown (**Figure 1**) in the agent-based GC model *hyphasma* (45–47). Based on the primary cause of GC shutdown, these mechanisms can be broadly categorized into four classes, namely, antigen limitation (M1-M4), Tfh help limitation (M5), increased exit from the GCs (M6) and decreased B cell division capacity (M7). Mechanisms M1-M4, result in antigen limitation in different ways such as the consumption by B cells, binding of soluble antibodies, FDC contraction and antigen internalization (**Figure 1**).

In simulations, we assume that Tfh signaling intensity is either dependent or independent of the amount of pMHC presented by GC B cells. In either assumption, when multiple B cells are interacting with a given Tfh cell, the Tfh cell polarizes and signals only the B cell with highest pMHC presentation (57). Due to such preferential polarization, there will be an indirect dependence of Tfh signals acquired on pMHC presentation even when the signaling intensity is considered to be independent of pMHC presented by GC B cells. Similarly, the number of GC B cell divisions is assumed to be dictated either by pMHC presentation (antigen captured) or by Tfh signals acquired. By combining these alternative determinants of Tfh signaling intensity and number of B cell divisions we consider four different sets of assumptions (**Table 1**). These alternate assumptions vary in the extent of dependence of Tfh signals and GC B cell divisions on pMHC presentation and are each supported by experimental observations. The scenario with direct dependence of Tfh signaling intensity on pMHC presented by GC B cells is based on the experimental observations suggesting that the magnitude of Tfh signals received depend on the pMHC presentation (5, 57). In the scenario with indirect dependence, the low affinity GC B cells are enabled to receive sufficient amount of Tfh signal in the absence of higher affinity clones, an observation that is also supported by experiments (58–60).

**Table 1:** Assumptions considered for the simulations.

Assumptions	Tfh signals	No. of divisions
A1	pMHC independent	pMHC dependent
A2	pMHC dependent	pMHC dependent
A3	pMHC independent	Tfh signal dependent
A4	pMHC dependent	Tfh signal dependent

pMHC, peptide-major histocompatibility complex; Tfh, T follicular helper cells.

In each mechanism, a control simulation was considered, where the GC does not shut down. This facilitates to infer the mechanistic changes during GC shutdown and also to identify mechanisms that alone are able to induce GC shutdown.

### Antigen Limitation (Mechanisms 1-4)

Antigen accessibility or availability was hypothesized as a mechanism of GC shutdown and various potential causes of antigen limitation have been identified (38, 43, 61). Firstly, antigen consumption by B cells leads to a small decrease in the antigen amount. We performed GC simulations to test whether this antigen decrease is able to shutdown GCs (**Figure S1**). Shutdown of GCs was observed irrespective of the assumptions in **Table 1** (**Figure S1A**), when the contribution of apoptosis and exit exceeds proliferation (**Figure S1B**). In the control simulations, antigen decrease due to B cell consumption was neglected, resulting in constant antigen amount throughout the simulation (**Figure S1C**) and therefore, the GC reaction did not terminate (**Figure S1A**, black curves). Varying the amount of antigen consumed by the B cell during a single encounter with the FDC, altered the speed of GC contraction (**Figure S1A**).

In this hypothesis, the average amount of antigen captured by B cells was reduced (**Figure S1D**) due to the decreased availability of antigen. The average amount of Tfh signals acquired per selected GC B cell increased due to alterations in affinity maturation and competition with other GC B cells. In assumptions A1 and A2, a decrease in the number of divisions was observed as it is assumed to depend on pMHC presentation. Alternatively, in assumptions A3 and A4, where the number of divisions is dependent on the Tfh signals acquired, the average number of divisions was not decreased but there was a small reduction in the fraction of selected LZ B cells, calculated as the fraction of LZ B cells in *Selected* state (**Figure S1D**, see methods). In these simulations, the entire fraction of selected LZ B cells was assumed to recycle to the DZ. Thus, a reduction in the fraction of selected GC B cells will reduce the fraction of GC B cells that recycle back to the DZ. Depending on the assumptions considered (see **Table 1**), decreased antigen uptake reduced the number of B cell divisions and/or the fraction of selected LZ B cells compared to the control simulation (**Figure S1D**). These results suggest that a small decrease in antigen amount is sufficient to cause GC contraction.

Similarly, binding of soluble antibodies can decrease antigen access of GC B cells by a process termed antibody feedback (38), which has been hypothesized to be involved in GC shutdown (38, 54). The strength of antibody feedback was varied using a scaling factor *N* that changes the concentration of antibodies promoting feedback (**Figure S2A**, see methods) and compared with a control simulation, where the soluble antibodies were not allowed to mask the antigen on FDCs. Antibody feedback led to GC contraction (**Figure S2B**), as the fraction of antigen masked by soluble antibodies increases (**Figure S2A**). This suggests that antibody feedback can independently shutdown GCs when the antibody concentration is high enough. Similar to the previous case, shutdown was due to a decrease in average antigen uptake of GC B cells that decreased the number of divisions and the fraction of selected LZ B cells compared to the control simulation (**Figure S2C**). For assumption A1, the number of divisions was decreased, and the fraction of selected LZ B cells increased. Only a decrease in the fraction of selected LZ B cells was observed in A3. Unlike in A1, the number of divisions and the fraction of selected LZ B cells were decreased for both A2 and A4. When the Tfh signaling intensity directly depends on pMHC presentation (A2 and A4), a decrease in Tfh signal acquisition was also observed (**Figure S2C**). As these simulations were performed with a single epitope, we tested whether the effects of antibody feedback depend on the antigen complexity by considering two epitopes in unequal proportions (90 % for immunodominant epitope and 10 % for rare epitope). Similar to the simulations with a single epitope, antibody feedback led to the contraction of GCs suggesting that the role of antibody feedback on GC shutdown persists even for antigens of higher complexity (**Figure S3**).

**Figure 2:**
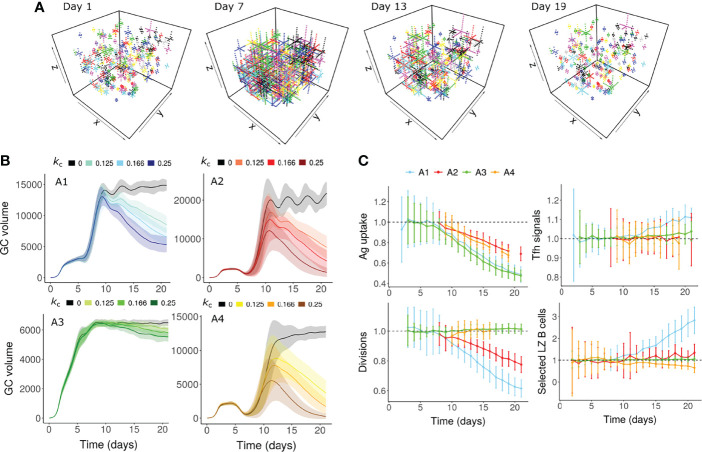
GC shutdown due to contraction of FDCs (Mechanism 3). **(A)** Visualization of the FDC network in a GC simulation at 4 different time points (Days 1, 7, 13 and 19). In this representative simulation, assumption A1 (**Table 1**) was used with an FDC contraction rate *k*_c_ of 0.166 µm per hour. Each lattice site occupied by the FDC is shown as a dot. Soma and dendrites belonging to the same FDC are shown in the same color. **(B)** GC dynamics with assumptions A1-A4. Black curves represent control simulations. Different colors represent different rates of FDC contraction *k*_c_ as shown in the labels above panel **(B)** Solid lines and shaded regions represent mean and standard deviation of 50 simulations, respectively. **(C)** Average antigen uptake per B cell, Tfh signals acquired per B cell, number of divisions of recycling GC B cells and fraction of Tfh selected LZ B cells. Readouts were normalized with that of the control simulation. Colors represent the assumptions A1-A4. Error bars represent standard deviation of 50 simulations. An FDC contraction rate of 0.166 µm per hour was used in panel **(C)** In all the panels, FDC extension rate was 0.166 µm per hour. GC, Germinal center; FDC, Follicular Dendritic Cell; Tfh, T follicular helper cell; Ag, Antigen; LZ, Light Zone.

The morphology of the FDC network changes during the GC reaction (2) and alterations in aged stromal cells impact the GC output (62). We hypothesized that a decrease in antigen accessibility due to changes in the area of FDC network might cause GC shutdown. We modelled dynamic changes in the FDC network by assuming that FDC dendrites extend at early stages of GC reaction and contract thereafter (**Figure 2A**). No contraction of FDC dendrites was considered in the control simulations. Contraction of FDCs was sufficient to cause GC shutdown in assumptions A1, A2 and A4 (**Figure 2B**). In A1 and A2, a decrease in antigen uptake of B cells reduced the average number of B cell divisions (**Figure 2C**). In A4, a small decrease in fraction of selected LZ B cells was observed. Under assumption A3, there were no observable changes in average divisions or fraction of selected cells (**Figure 2C**). Consequently, there was only a small reduction in GC volume (**Figure 2B**). This implies that when the number of divisions and the Tfh signaling intensity are not directly dependent on pMHC presentation of B cells, the impact of FDC contraction is weak and is unlikely to cause GC shutdown independently.

ICs in FDCs undergo a protective cycling mechanism (56, 63). Factors determining cycling rates are currently unknown but a modulation in IC cycling rate constants might be expected during the GC reaction (55). By the dynamic modulation of IC externalization rate constant, we tested whether a decrease in surface IC amount due to changes in cycling rate constants can shut down GCs (**Figure S4A**). This led to a decrease in surface antigen amount on FDCs (**Figure S4A**) and was able to shutdown GCs in all the assumptions (**Figure S4B**). Alterations in antigen uptake of B cells impacted either the number of divisions or the number of selected LZ B cells (**Figure S4C**).

Collectively, M1-M4 suggest that changes in antigen accessibility or availability have the potential to act as a primary mechanism of GC shutdown.

### Tfh Help Limitation (Mechanism 5)

**Figure 3:**
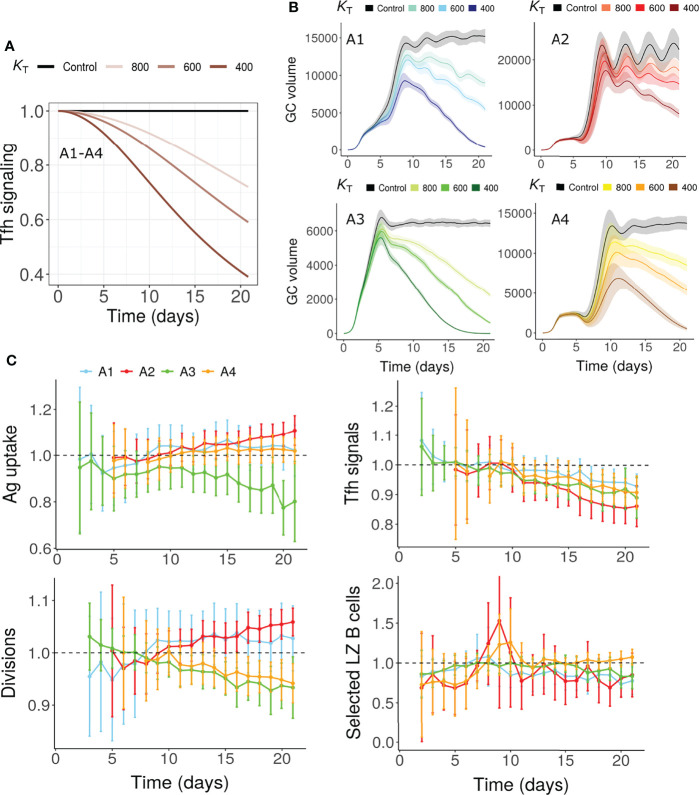
GC shutdown due to changes in Tfh signaling intensity (Mechanism 5). **(A)** Maximum Tfh signaling intensity *T*_max_ *vs* time according to Equation 8 in assumptions A1-A4. Values were normalized to the maximum signaling intensity at the start of the simulation. Colors represent different values of *K*_T_ and black curve labelled control represents the control simulation where no decrease in Tfh signaling intensity was considered. *K*_T_ is the time point in hours where the maximum Tfh signaling intensity decreases to half of its initial value (see Equation 8, Methods sections). **(B)** GC dynamics in assumptions A1-A4. Black curves represent control simulations. Different colors represent value of *K*_T_ used in Equation 8 and are shown in labels above panel **(B)** Solid lines and shaded regions represent mean and standard deviation of 50 simulations, respectively. **(C)** Average antigen uptake per B cell, Tfh signals acquired per B cell, average number of divisions per recycling GC B cell and fraction of Tfh selected centrocytes (with *K*_T_ = 600). Readouts were normalized with that of the control simulation. Colors represent the assumptions A1-A4. Error bars represent standard deviation of 50 simulations. GC, Germinal center; Tfh, T follicular helper cell; Ag, Antigen; LZ, Light Zone.

As Tfh cells undergo progressive changes in the GCs (31, 32), we tested whether changes in Tfh signaling capacity could terminate GCs in the absence of antigen limitation. More specifically, due to the suppressive nature of Tfr cells in the GCs (34, 64–68), a progressive decrease in Tfh signaling intensity was assumed in this hypothesis (**Figure 3A**). This mechanism was also able to terminate GCs (**Figure 3B**). As in the antigen limitation models, a reduction in number of divisions and fraction of selected LZ B cells were observed depending on the assumptions considered (**Figure 3C**). For A1 and A2, the number of divisions was not reduced unlike in the antigen limitation models M1-M4. However, a decrease in the number of divisions was seen for A3 and A4. Therefore, changes in the Tfh help intensity in the absence of antigen limitation is also a possible mechanism of GC shutdown. Due to the impact of this mechanism on GC B cell affinity maturation and selection, it also affected antigen uptake of GC B cells in dependence on assumptions A1-A4 (**Figure 3C**).

### Increased Terminal Differentiation (Mechanism 6)

**Figure 4:**
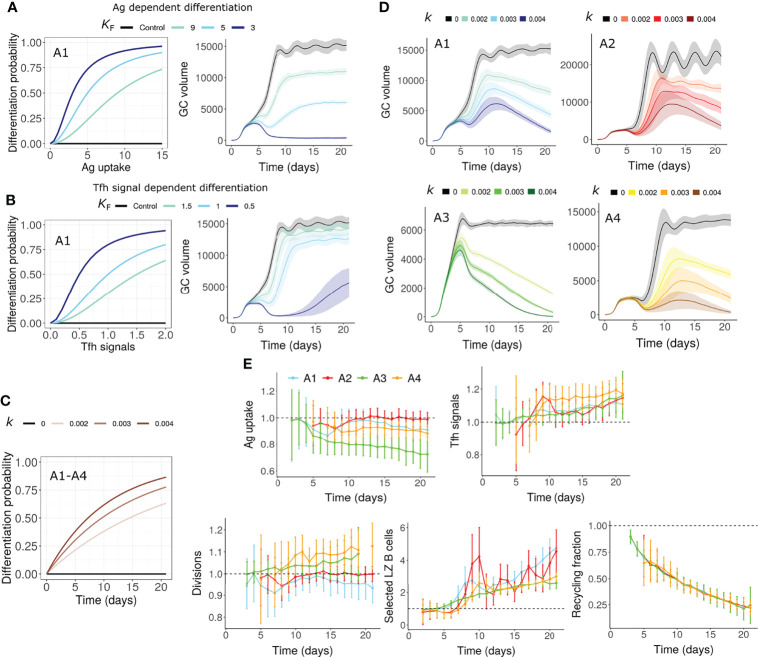
GC shutdown due to terminal differentiation of B cells (Mechanism 6). **(A, B)** Differentiation probability in antigen dependent **(A)** and Tfh signal dependent **(B)** terminal differentiation and corresponding GC volume dynamics. Colors represent different values of *K*_F_ in Equation 9. *K*_F_ is the amount of Ag captured (in antigen dependent terminal differentiation) or Tfh signals received (in Tfh signal dependent terminal differentiation) for half-maximal differentiation probability (see Methods). Assumption A1 was considered in these simulations. **(C, D)** Terminal differentiation probability *vs* time according to Equation 10 in assumptions A1-A4 for time dependent terminal differentiation **(C)**. Colors represent different values of *k* and corresponding GC dynamics **(D)**. Black curves represent control simulations. Different colors represent value of *k* in Equation 10 and are shown in labels above **(C, D)** Value of *k* controls the increase in differentiation probability. Solid lines and shaded regions in panels A, B and D represent mean and standard deviation of 50 simulations, respectively. **(E)** Average antigen uptake per B cell, Tfh signals acquired per B cell, average number of divisions per recycling GC B cell and fraction of Tfh selected LZ B cells for time dependent terminal differentiation (*k* = 0.003). Readouts were normalized with that of the control simulation. Colors represent the assumptions A1-A4. Error bars represent standard deviation of 50 simulations. GC, Germinal center; Tfh, T follicular helper cell; Ag, Antigen; LZ, Light Zone.

Terminal differentiation of GC B cells and exit from the GCs may also act as the cause of GC shutdown. Although the exact mechanism governing the differentiation of memory and plasma cells is not known, the strength of Tfh-B cell interaction has been shown to determine the selected GC B cell fate (6). We assumed that selected LZ GC B cells differentiate into output cells (memory or plasma cells) with a probability that is dependent on antigen uptake or Tfh signals received (**Figures 4A, B**). Therefore, only a fraction of selected LZ B cells recycles to the DZ unlike other mechanisms where all selected B cells recycle to the DZ. Unexpectedly, under all the assumptions considered (A1-A4), either the GC could not be terminated or underwent premature termination at a very early stage (**Figures 4A, B** for A1). Hence, terminal differentiation when solely dependent on antigen uptake or Tfh signals is unlikely to cause GC shutdown in the absence of other mechanisms that impact antigen uptake or Tfh signals. Alternatively, it is possible that the terminal differentiation depends on other unknown factors.

To account for the unknown factors, we assumed that the probability of differentiation into output cells is time dependent (**Figure 4C**). In this case, there was a progressive increase in the terminal differentiation of GC B cells and shutdown of GCs were observed (**Figure 4D**). We quantified the recycling fraction as the fraction of selected LZ B cells that recycle to DZ. A large reduction in the fraction of recycling cells was observed in this mechanism due to increased exit from the GCs (**Figure 4E**). As the direct dependence of differentiation on antigen uptake and Tfh signals acquired was ignored, the impact on the number of divisions was weak (**Figure 4E**).

### Limited B Cell Division Capacity (Mechanism 7)

Metabolic changes occur in B cells during the GC reaction. For instance, the dependence of GC B cells on exogenously supplied fatty acids varied at different time points of the GC reaction likely due to the exhaustion of fatty acids reserves in late GC B cells (69). Considering this finding, we presume that such progressive changes in GC B cells might lead to decreased division capacity irrespective of antigen uptake of GC B cells.

**Figure 5:**
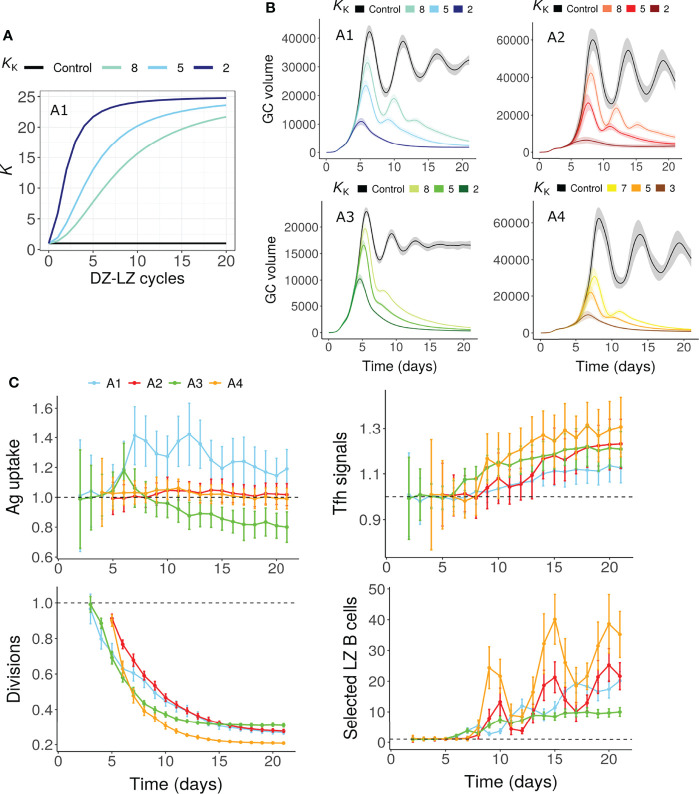
GC shutdown due to limited B cell division capacity (Mechanism 7). **(A, B)** *K vs* number of DZ-LZ cycles according to Equation 11 **(A)** and GC dynamics **(B)** for assumptions A1-A4. Colors represent different values of *K*_K_ as shown above the panels and black curves represent the control simulation. *K*_K_ controls the increase in *K* with increasing number of DZ-LZ cycles. The value of *K* determines the dependence of the number of divisions on the amount of antigen captured in assumptions A1/A2 or Tfh signals acquired by B cells in A3/A4 (see Methods). Solid lines and shaded regions represent mean and standard deviation of 50 simulations, respectively. **(C)** Average antigen uptake per B cell, Tfh signals acquired per B cell, average number of divisions per recycling GC B cell and fraction of Tfh selected LZ B cells (with *K*_K_ = 5). Readouts were normalized with that of the control simulation. Colors represent the assumptions A1-A4. Error bars represent standard deviation of 50 simulations. GC, Germinal center; Tfh, T follicular helper cell; Ag, Antigen; DZ, Dark zone; LZ, Light zone.

To mimic this scenario, the division capacity of GC B cells was assumed to decrease due to continuous circulation between the GC zones. Therefore, the number of divisions was assumed to depend on the number of DZ-LZ cycles underwent by the GC B cell, in addition to the amount of antigen captured or the Tfh signals acquired (**Figure 5A**, see methods). This mechanism led to a decreased number of divisions over time leading to GC shutdown (**Figures 5B, C**) even in the absence of alterations in antigen amount on FDCs or signaling ability of Tfh cells. The fraction of selected LZ B cells and Tfh signals acquired per B cell were not reduced (**Figure 5C**).

### Experiments Proposed

Results discussed so far clearly show that mechanisms targeting various processes in the GCs can promote shutdown. Based on the *in-silico* predictions, potential experiments are suggested in this section to test the proposed mechanisms.

#### Density of pMHC Presentation

**Figure 6:**
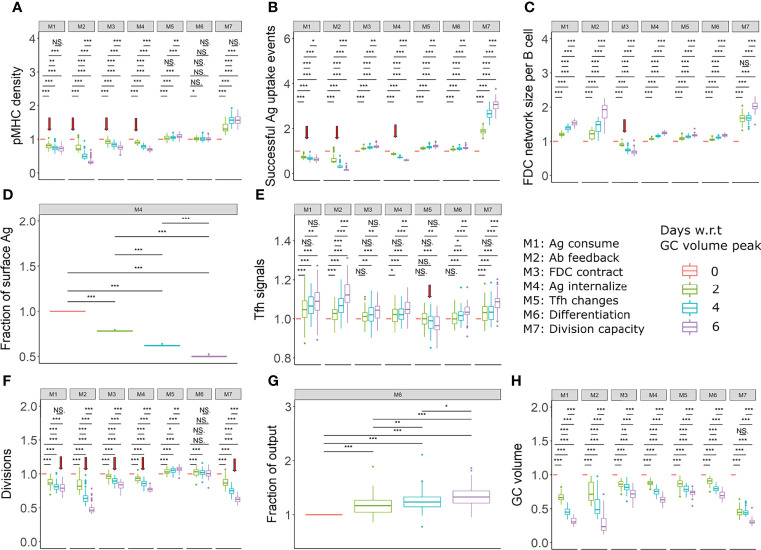
Proposed experiments to identify the existence of mechanisms (in assumption A1). **(A)** pMHC density of selected centrocytes, **(B)** Fraction of successful antigen uptake events among all FDC-B cell encounters, **(C)** FDC network size per B cell, **(D)** Fraction of Ag on FDC surface, **(E)** Tfh signals received by selected centrocytes, **(F)** Average number of divisions of recycling centrocytes, **(G)** Fraction of output cells among Tfh selected cells, **(H)** GC volume in all mechanisms. All readouts were normalized with respect to (w.r.t) the value at the peak of the GC reaction. Different colors represent the different time points with respect to GC reaction peak. The FDC network size per B cell was calculated by dividing the total number of lattice sites occupied by FDCs by the total number of GC B cells. Statistical tests were performed by Wilcoxon test. Error bars represent standard deviation of 50 simulations. Red arrows indicate a decreasing trend in readouts that differs from other mechanisms. Parameter values used in different mechanism: M1: 1 unit of Ag consumption per FDC-B cell interaction, M2: *N*=300, M3: FDC contraction rate = 0.166 µm per hour, M4: *K*_ext_ =150, M5: *K*_T_ = 600, M6: *k* = 0.003, M7: *K*_K_ = 5. GC, Germinal center; Tfh, T follicular helper cell; Ag, Antigen; Ab, Antibody; FDC, Follicular dendritic cell. * = p < 0.05, ** = p < 0.01, *** = p < 0.001. NS, not significant.

Average antigen uptake of GC B cells decreases over time due to the mechanism of antigen limitation. Therefore, a decrease in the density of pMHC presentation is expected to be associated with GC contraction in mechanisms M1-M4 and might help indicate the existence of antigen limitation. The decrease was confirmed *in silico* for mechanisms M1-M4 and was not found for mechanisms M5-M7 (**Figures 6A**, **S5-S7A**). The observed trend in the presented pMHC density depended on the characteristics of the GC volume kinetics: In mechanism M2 for assumptions A1, A2 and A4, there was an immediate decrease in pMHC density after the GC attained a maximum size. However, for assumption A3, the decrease in pMHC density was delayed after the peak GC volume was first attained and a closer to maximum GC volume was maintained for a longer period (**Figure S6A**). Given that the GC kinetics is sensitive to immunization conditions and experimental setups, the GC volume might not always decline immediately after a maximum size is attained and there is a need to measure the GC volume in parallel with pMHC density to identify the GC contraction period. This suggests that a potential way to differentiate the presence of antigen limitation from other mechanisms is to quantify the density of pMHC presentation at different time points after the peak of the GC reaction and during GC contraction. As antigen uptake involves BCR crosslinking that induces changes in phosphorylation of Foxo1 (9), antigen uptake of GC B cells might be measured by such BCR crosslinking induced signaling changes. The experimental observation of a decrease in pMHC presentation over time during GC contraction would indicate the importance of at least one of the antigen limitation mechanisms M1-M4 for GC shutdown, although it doesn’t exclude the co-existence of mechanisms M5-M7. In contrast, if pMHC presentation was found to increase over time or to remain constant, this would support mechanisms M5-M7 to be responsible of GC shutdown.

#### Fraction of Successful Antigen Acquisition Events

In the FDC contraction mechanism (M3), antigen limitation is due to a reduced number of B cell- FDC encounters. However, in the other antigen limitation mechanisms there is a decrease in the number of B cell-FDC encounters with successful antigen capture (**Figures 6B**, **S5-S7B**). Quantifying the fraction of FDC encounters with successful antigen acquisition can help identify the cause of decreased antigen access and can differentiate the FDC contraction mechanism from other antigen limiting mechanisms.

FDC-B cell interactions and antigen capture of naïve B cells in primary follicles have been visualized by two-photon microscopy with Phycoerythrin-labelled antigen and CFSE/CFP labelled B cells (70). A similar analysis monitoring the fraction of FDC encounters resulting in successful antigen capture needs to be performed at different time windows spanning the GC reaction. Provided that antigen limitation is a critical mechanism of GC shutdown, an increasing fraction of successful antigen uptake events over time would support the FDC contraction mechanism M3 while a decreasing fraction would support M1, M2 or M4.

#### FDC Network Size per B Cell

The FDC network size per B cell was calculated by dividing the total number of lattice sites occupied by the FDC network by the total number of GC B cells, which can be interpreted as a measure for the GC space occupied by FDCs.

In the absence of FDC contraction, the FDC network size per B cell decreases until the GC volume attains a peak and increases thereafter. However, in the presence of FDC contraction, the FDC network size per B cell decreases or remains constant after the peak of the GC reaction (**Figures 6C**, **S5–S7C**). The FDC network can be visualized by staining for complement receptor CD35 (70). Similar labelling and measurement of the FDC network size per B cell would be able to detect changes in the FDC morphology. A reduced or constant FDC network size per B cell after the peak of the GC volume would indicate the contraction of FDCs while an increase in FDC network size per B cell would exclude this mechanism.

#### FDC Antigen Distribution

The IC cycling rate constants determine the fraction of ICs on the FDC surface which is defined as the ratio of IC amount on the FDC surface, and the total antigen amount retained in the FDC. The fraction of ICs on the FDC surface is expected to remain constant if the IC cycling rate constants are constant throughout the GC reaction. Modulations of IC cycling rate constants as proposed in mechanism M4 will lead to differences in the distribution of ICs on the FDC’s surface and interior during the GC reaction such that the fraction of ICs on the FDC surface decreases over time (**Figures 6D**, **S5-S7 D**). This can be observed by monitoring the amount of antigen on the FDC’s surface and interior at multiple time points of the GC reaction. Normalization of surface antigen amount with the total antigen amount in FDCs would be necessary as there would be a decrease in the total antigen amount due to consumption by B cells or other reasons. Even though IC cycling was not considered in mechanisms other than M4, it is expected that the fraction of ICs on the FDC surface will remain constant in other mechanisms in the presence of IC cycling with a constant rate constant. Thus, measuring the distribution and localization of antigen on the FDC surface at different stages of the GC reaction can be used as a test for the existence of changes in IC cycling rate constants. A decrease in the fraction of ICs on the FDC surface during the GC reaction would suggest an increased IC internalization while a constant fraction of ICs on the surface would indicate a lack of modulation of the IC cycling rate constant.

#### Decreased Tfh Signals

In mechanism M5, due to reduced Tfh signaling, a decrease in Tfh signals acquired by GC B cells was observed over time (**Figures 6E**, **S5-S7E**). Expression of Myc is proportional to the strength of Tfh signaling (71). Therefore, c-Myc levels might act as a proxy for Tfh signals in the simulations. c-Myc expression levels of c-Myc+ GC B cell subpopulations have been previously examined (72). Similar analysis has to be performed at multiple time points to detect any decrease in Tfh signaling strength during GC contraction.

However, in assumptions A1 and A4, only a subtle decrease in Tfh signals was seen that was not statistically significant. In addition, a decrease in Tfh signals is also seen in the case of antigen limitation models depending on the assumptions (**Figures S5E, S7E**). Therefore, it should be noted that the measurement of Tfh signal acquisition might not always be conclusive. A decrease in Tfh signals combined with an increased or constant pMHC density would support M5, but a decrease in pMHC density or the absence of a significant decrease in Tfh signals do not necessarily exclude M5.

#### Quantifying Average Divisions

A decrease in the average number of divisions is observed in many mechanisms including antigen or Tfh help limitation, and reduced B cell division capacity (**Figures 6F**, **S5-S7F**). Due to this, mechanisms proposed in this study cannot be distinguished easily by measuring the average number of divisions over time. However, a decrease in the average number of divisions combined with an absence of changes that indicate the existence of other mechanisms as shown in **Figures 6**, **S5-S7** suggest the presence of mechanisms that affect the B cell division capacity directly, as in M7 (**Figures 6F**, **S5-S7F**). Previously, a transgenic strategy with tTA-H2B-mCh (transactivator (tTA) protein and histone H2B-mCherry fusion protein) was used to monitor the number of GC B cell divisions (73).

#### Fraction of Cells With Differentiation Markers

When the GC shutdown is based on increased exit of cells due to differentiation, an increase over time in the fraction of Tfh selected cells with differentiation markers can be expected when quantified around the peak of the GC reaction (**Figures 6G**, **S5-S7G**). Precursors of memory and plasma cells have been shown to specifically express markers CCR6 (7) and BLIMP-1 (74), respectively. A combination of markers including CXCR4, CD86, CD69, CD23 and CD21/35 have been shown to distinguish potential output cells from recycling B cells in a heterogenous c-Myc+ GC B cell population (72). Quantifying the fraction of cells with these markers can be considered as a test for increased terminal differentiation and exit mechanism. No experimentally observed increase in the fraction of output cells after the GC volume peak would exclude this mechanism M6 to contribute to GC shutdown.

### Implications on GC Output

In general, GC shutdown decreases the total output production and extent of affinity maturation. We tested whether any of the shutdown mechanism accelerated affinity maturation or produced more output cells within a restricted period of time. Changes in quantity and quality of output were largely dependent on the assumptions considered (**Figure S8**) and were also sensitive to the strength of shutdown stimulus (not shown).

**Figure 7:**
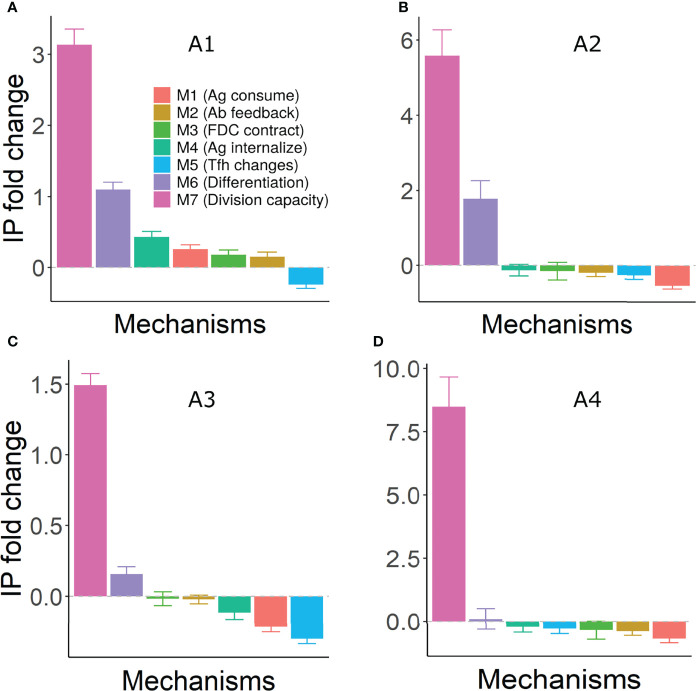
Fold change in efficiency (IP) of GC reaction under different mechanisms of shutdown. Panels **(A–D)** represent assumptions A1-A4, respectively. IP was calculated using equation 13 at day 21 of the GC simulation and fold change was calculated with respect to the IP of the corresponding control simulation. Error bars represent standard deviation of 50 simulations. Positive and negative values represent an increase and decrease, respectively, compared to the control simulation. Parameter values used in different mechanism: M1: 1 unit of Ag consumption per FDC-B cell interaction, M2: *N*=300, M3: FDC contraction rate = 0.166 µm per hour, M4: *K*_ext_ =150, 200, 80 and 250 in assumptions A1-A4, M5: *K*_T_ = 600, M6: *k* = 0.003, M7: *K*_K_ = 5. GC, Germinal center; Tfh, T follicular helper cell; Ag, Antigen; Ab, Antibody; FDC, Follicular dendritic cell; IP, Immune Power.

To combine the effects of changes in quality and quantity, efficiency of GC reaction was estimated by a quantity termed immune power (IP) that mimics an ELISA test (53, 54). Mechanisms M6 and M7 (increased terminal differentiation and decreased B cell division capacity) led to an increased GC efficiency suggesting that these mechanisms of shutdown would be beneficial in accelerating output production and antibody responses under most assumptions (**Figure 7**). On the other hand, mechanism M5 (Tfh signal changes) consistently had a suppressive effect. For antigen limiting mechanisms M1-M4, an increase in GC efficiency was seen only under assumption A1 (**Figure 7**).

## Discussion

Although GC shutdown is an important determinant of the quality and quantity of antibody responses, mechanisms of GC shutdown are poorly understood. Premature GC shutdown can impair the protection against invading pathogens or reduce the efficacy of vaccinations and lack of GC shutdown has the potential to give rise to GC-derived B cell lymphomas. Thus, a better understanding of GC shutdown has a wide range of applications from enhancing antibody responses to blocking the progression of B cell lymphomas and ectopic GCs. In this study, we tested potential mechanisms of GC shutdown *in-silico*, to identify the most promising target mechanisms that are self-sufficient to contract GCs. To facilitate experimental analysis of these mechanisms, we suggest potential experimental setups based on the *in-silico* predictions.

In all of the mechanisms identified, from antigen limitation to reduced B cell division capacity, GC shutdown was ultimately caused by a decrease in either the number of B cell divisions or reduced recycling to the DZ or both. Gitlin et al., demonstrated that GC B cell divisions are proportional to antigen uptake and pMHC presentation to Tfh cells (73). But, the exact relationship between pMHC presentation and number of divisions was only approximated in computer simulations based on data about the ratio of DZ and LZ B cells (47), as the mechanistic details linking antigen capture and number of divisions are only partly understood. Tfh cells induce Myc transcription factor in GC B cells in direct proportion to the amount of antigen captured (71). But as the Myc expression is transiently downregulated, subsequent activation of CyclinD3 is important (75), that controls the number of cell divisions in a dose-dependent way (76). Post-transcriptional regulation of Myc transcripts by epigenetic modulators may also affect the B cell division capacity by controlling the stability of Myc transcripts (77). A better understanding of factors that control GC B cell divisions and factors inducing cell cycle re-entry would be highly beneficial for a mechanistic understanding of GC shutdown. In view of M7, whether the ability of GC B cells to proliferate decreases after a long period of stay in the GC due to exhaustion or limited energy supply also needs to be investigated. GC shutdown due to reduced B cell division capacity optimizes the immune power and would be a rather efficient way of limiting the duration of the GC response, thus, also limiting potential dysregulations.

Furthermore, outcome of the shutdown mechanisms was sensitive to the set of assumptions considered for the determinants of Tfh signal intensity and the number of B cell divisions. Although there is considerable evidence for the dependence of productive B-Tfh cell interactions on pMHC presentation (5), the extent of dependence is unclear. It has been suggested that altering the pMHC presentation does not alter the competitive fitness and certain extent of permissiveness is observed in the selection process that allows the low affinity B cells to persist in the GCs (58, 59). An unproven hypothesis reconciling those observations is that Tfh signaling adapts its intensity in dependence of experienced levels of pMHC presentation (11). As the four sets of assumptions considered (A1-A4) appear consistent with present experimental observations, the most realistic among these could not be determined. This suggests a need for future studies in clarifying these assumptions under different experimental settings.

Different mechanisms altering antigen availability or accessibility were identified as self-sufficient mechanisms of GC shutdown. Ability of antigen consumption by B cells to cause GC shutdown varied depending on the amount of antigen captured by B cells relative to the total antigen amount on FDCs. Therefore, consumption of antigen by GC B cells has a great potential to terminate GCs at least under antigen limiting conditions, without the need to exhaust the total antigen amount on FDCs. Further, we suggested numerous ways in which changes in antigen accessibility might occur despite the presence of sufficient antigen in the FDC network. *In-silico* simulations suggested the potential of antibody feedback in terminating GCs. Antibody feedback as a mechanism of GC shutdown is an attractive option because it self-regulates the necessity of continuation of the GC response by monitoring success of the GC reaction right in the GC area. Dynamic changes in FDC network by the contraction of FDCs (2) was capable of contracting GCs and might be tested by the measurement of FDC network size per B cell. We speculated that the FDC-IC cycling rate constants is modulated during GC reaction and this mechanism could terminate GCs. Modulation of IC cycling rate constant could be tested by the measurement of the fraction of FDC-ICs displayed on the FDC surface. These results support the possibility that antigen limitation might be a major factor involved in GC shutdown in the case of immunization with non-replicating agents. Therefore, testing for the signs of antigen limitation could be considered as a first step in the analysis of GC shutdown mechanisms. Decreased antigen uptake of GC B cells in any of the antigen limiting mechanisms is expected to be reflected in the density of pMHC presentation, and a time course analysis of pMHC density at different stages of GC reaction might help identify the presence of antigen limitation. Differentiating the effects of antigen limitation due to antigen consumption of B cells versus antigen masking by soluble antibodies could be technically challenging and needs to be addressed in the future.

Similar to the slow decrease in antigen by B cell consumption, natural decay of antigen or clearance from the surface of FDCs might also play a role in GC shutdown. The rate of decrease might vary depending on the nature of antigen and might contribute to the differences in GC lifetime observed with different antigens (15–17). Apart from B cells, other cell types such as tingible body macrophages can uptake antigen from FDCs (78) but the kinetics of antigen uptake and its role in GC shutdown is unclear. Tingible body macrophages have also been shown to affect the magnitude of GC reaction by promoting the clearance of apoptotic cells (79). The aforementioned mechanisms are expected to differ in their contribution to antigen limitation based on their specific antigen clearance kinetics. Changes in the morphology of FDCs or iccosome organization (2, 61) without changes in the FDC network area might also be expected. This needs more detailed investigation of the organization of ICs on the FDC surface. In addition to the capture of ICs from FDCs, B cells also acquire surface markers present on FDCs such as the CR1 and BP-3 suggesting other forms of information transfer (70). The significance of such transfer is unknown and might be of importance in the context of GC shutdown. Consistent with our findings, experimental observations have shown that GC reactions cannot be sustained in the presence of a defect in long-term antigen retention (80, 81). On the contrary, it has been shown that GC reactions are not severely affected in the absence of ICs on FDCs (82). It remains to be investigated whether the importance of FDC trapped ICs varies under different conditions.

Tfh signal limitation might occur due to the suppressive action of Tfr cells although the role and mechanism of Tfr action in the GCs is not entirely understood (83). Hence, understanding the precise role of Tfrs is important to test their influence on GC shutdown. On the contrary, Tfh cells have been shown to undergo selection (31) which might increase the signals delivered to B cells at late time points. Whether the suppressive activity of Tfr could overcome the potential increase in Tfh signals due to such selection process needs to be addressed. As we have found a comparably weak immune power of the GC reaction if shutdown is based on reduced Tfh signaling, this might be considered as an evolutionary argument against this mechanism of GC shutdown.

The strength of GC B cell and Tfh cell interactions has been shown to determine the fate decisions between recycling to DZ or terminal differentiation (6). The exact combination of signals underlying the differentiation of GC B cells into plasma or memory cells is unclear. But on a phenomenological level, an increased exit of GC B cells with high antigen uptake or Tfh signals, failed to terminate GCs *in silico* (**Figure 4**). However, it is possible that the differentiation into plasma cells/memory cells is more complex and needs to be better understood. Differentiation probability of GC B cells has been estimated to be 0.2 or lower (18) based on experimental constraints. In this study, we have assumed that the differentiation probability progressively increases over time in the terminal differentiation mechanism M6. This mechanism was able to promote GC shutdown and can be tested by measuring the fraction of GC B cells expressing differentiation markers at different timepoints of the GC reaction. An *in silico* modeling study (84) predicted that a combination of asymmetric division and affinity-based Tfh signaling can explain the temporal switch in memory to plasma cell differentiation (85). Such studies unravelling the differences in the fates of GC B cells – recycling *vs* plasma/memory cell differentiation is also relevant to understand GC shutdown. GCs with extended lifetime are observed in Peyer’s patches and during viral infections. Such GCs differ from conventional GCs and are characterized by persistent antigen deposition, re-seeding of new B cell clones, alterations in the Tfh/Tfr ratio, and in the functional states of FDCs, Tfhs and Tfrs (86–90). Adjuvants can also enhance the lifetime of GC responses (91) and have been shown to act on dendritic cells/B cells (92), increase the deposition of antigen on FDCs (93) and alter the Tfh/Tfr ratio (94). All of the mechanisms proposed in this study appear consistent with the extended nature of GCs observed under these conditions. For instance, persistent antigen deposition and persistent entry of Tfh could overcome antigen limitation and Tfh signal limitation, respectively, and increase the longevity of GCs. Further, persistent entry of new cells can counteract the increased B cell exit from the GCs and might replenish a limited division capacity in the GCs. Multiple interconnected factors are involved in the GC reaction such as the antigen collection by B cells, interaction of B cells with Tfh cells and subsequent B cell divisions. The outcome of the GC simulations for each shutdown mechanism is a collective effect of these interconnected factors. The in-silico approach has the advantage that we can investigate individual mechanisms of GC shutdown even when embedded in an interconnected network of mechanisms. It is likely that many mechanisms act together to promote GC shutdown and that the contribution of different mechanisms might vary under different conditions. When a combination of the features discussed in this study (**Figure 6**) for different mechanisms is observed, this would indicate the action of multiple mechanisms. Whether the proposed changes in Tfh signaling capacity, terminal differentiation and division capacity of B cells could arise as a consequence of antigen limitation is unclear. A reduction in the number of antigen uptake events by GC B cells was also observed in the absence of limiting antigen amount, as antigen acquisition of GC B cells are altered due to secondary reasons such as changes in affinity maturation or competition for FDC binding sites.

Although, a longer GC lifetime is generally expected to increase plasma cell production and affinity maturation in a long term, we showed that GC shutdown due to increased terminal differentiation and limited B cell division capacity were able to accelerate the production of output cells and affinity maturation, thus increasing the efficiency of GC reactions. Depending on the mechanism of shutdown, the impact on output cell production and affinity varied, suggesting that different targets to alter the GC lifetime might have differing effects on the productivity of GCs. As a longer GC lifetime also increases the possibility of dysregulation, it might be speculated that there would be a tradeoff between efficient protection from infection and minimizing the emergence of potential dysregulations/self-reactivity.

## Data Availability Statement

The datasets presented in this study can be found in online repositories. The names of the repository/repositories and accession number(s) can be found below: Code and scripts used in this study are available at Zenodo: https://doi.org/10.5281/zenodo.6395396.

## Author Contributions

TA, SB, and MM-H designed the study. TA performed simulations. SB and MM-H supervised the project. TA, SB, and MM-H wrote the manuscript. All authors contributed to the article and approved the submitted version.

## Funding

TA was supported by the European Union's Horizon 2020 research and innovation programme COSMIC under the Marie Sklodowska-Curie grant agreement no. 765158.

## Conflict of Interest

The authors declare that the research was conducted in the absence of any commercial or financial relationships that could be construed as a potential conflict of interest.

## Publisher’s Note

All claims expressed in this article are solely those of the authors and do not necessarily represent those of their affiliated organizations, or those of the publisher, the editors and the reviewers. Any product that may be evaluated in this article, or claim that may be made by its manufacturer, is not guaranteed or endorsed by the publisher.
