# Supplementary material for: Investigating the Mechanism of Germinal Center Shutdown
Source: Front Immunol. 2022 Jul 14;13:922318. doi: 10.3389/fimmu.2022.922318 (PMC9329532; doi:10.3389/fimmu.2022.922318)
Supplement: Supplementary file 1 [file DataSheet_1.docx]

Supplementary Material

# Supplementary Figures


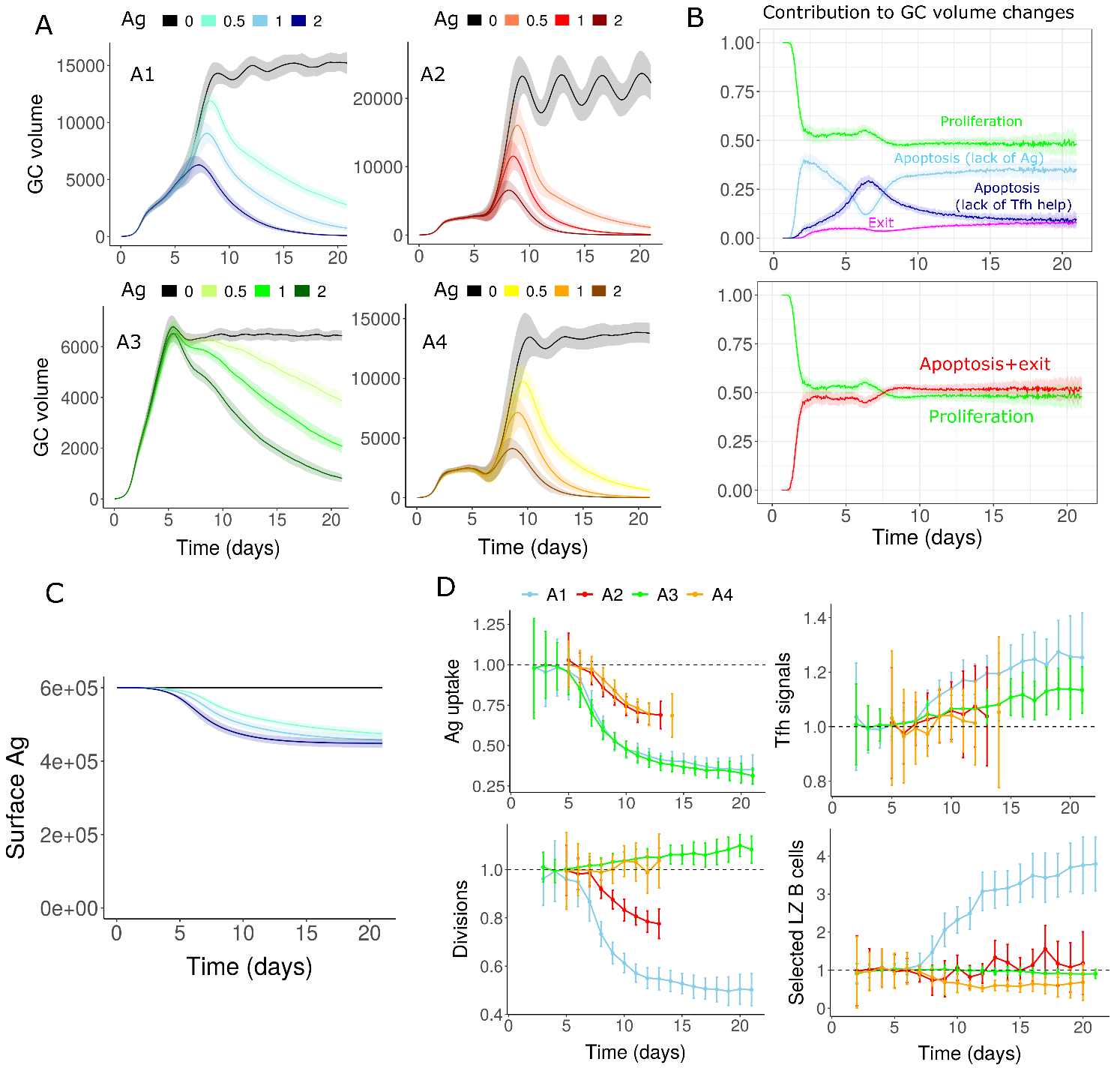


**Figure S1: GC shutdown due to B cell consumption of antigen (Mechanism 1).** A) GC dynamics under assumptions A1-A4 (Table 1). Black curves represent control simulations. The color represents the number of antigen portions consumed per encounter with FDCs as shown in the title of panel A. B) Contribution of proliferation, apoptosis and exit to GC volume changes for assumption A1. C) Total antigen amount on FDC surface for assumption A1. In panels A, B and C, solid lines and shaded regions represent mean and standard deviation of 50 simulations, respectively. D) Average antigen uptake per B cell, average Tfh signals acquired per B cell, average number of divisions per recycling GC B cell and fraction of Tfh selected LZ B cells. Readouts were normalized with that of the control simulation. Colors represent the assumptions A1-A4. Error bars represent standard deviation of 50 simulations. In panels B and D, antigen consumed per FDC-B cell encounter is 1. GC: Germinal center; FDC: Follicular dendritic cell; Tfh: T follicular helper cell; LZ: Light Zone; Ag: Antigen.


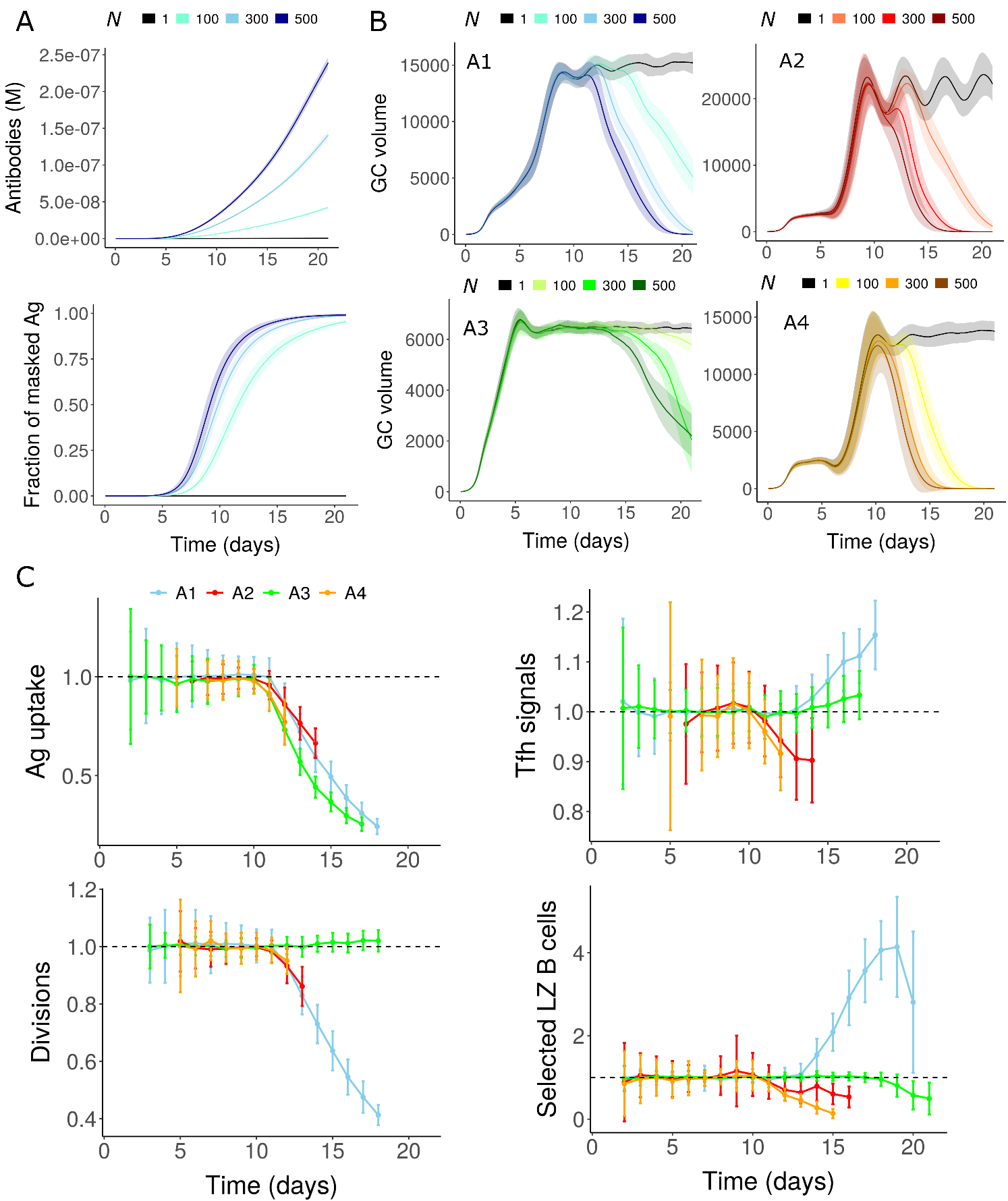


**Figure S2: GC shutdown due to antibody feedback (Mechanism 2).** A) Concentration of antibodies promoting feedback and fraction of masked antigen on FDCs for assumption A1. B) GC dynamics under assumptions A1-A4. Black curves represent control simulations. Different colors represent the value of the scaling factor *N* used to vary the strength of antibody feedback (see Equation 5) as shown in the labels above panel B. In panels A and B, solid lines and shaded regions represent mean and standard deviation of 50 simulations, respectively. C) Average antigen uptake per B cell, Tfh signals acquired per B cell, number of divisions of recycling GC B cells and fraction of Tfh selected LZ B cells. Readouts for *N*=300 were normalized with that of the control simulation. Colors represent the assumptions A1-A4. Error bars represent standard deviation of 50 simulations. GC: Germinal center; FDC: Follicular dendritic cell; Tfh: T follicular helper cell; LZ: Light Zone; Ag: Antigen.


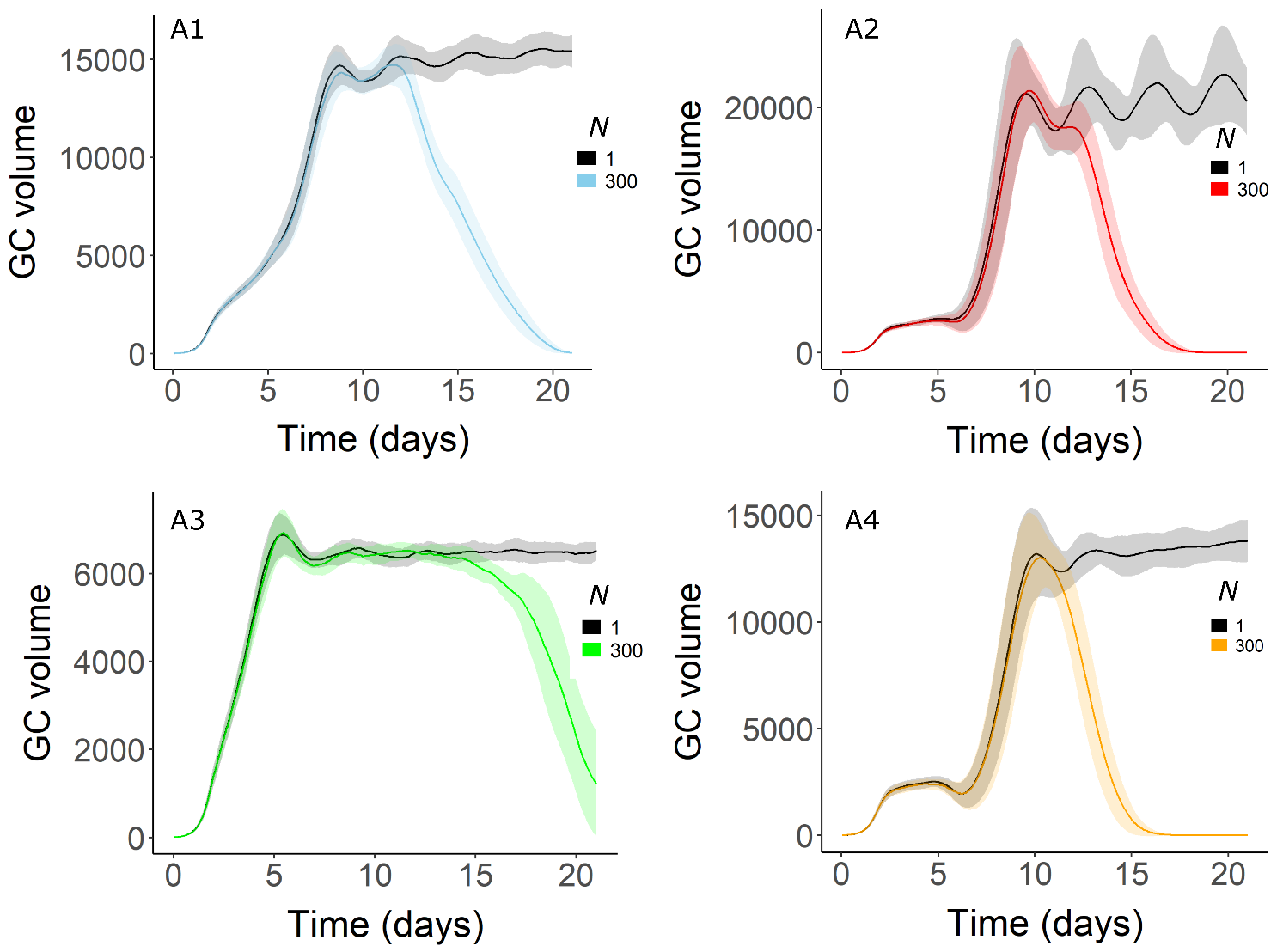


**Figure S3: GC shutdown due to antibody feedback (Mechanism 2) as in Figure S2 but with two epitopes.** Two epitopes were considered in unequal proportions (Immunodominant epitope: Ag 1, shape space position 3333, percentage of total antigen amount: 90 %; Rare epitope: Ag 2, shape space position 5555, percentage of total antigen amount: 10 %). GC dynamics under assumptions A1-A4. Black curves represent control simulations. Scaling factor *N*=300 was used to vary the strength of antibody feedback (see Equation 5). Solid lines and shaded regions represent mean and standard deviation of 30 simulations, respectively. GC: Germinal center.


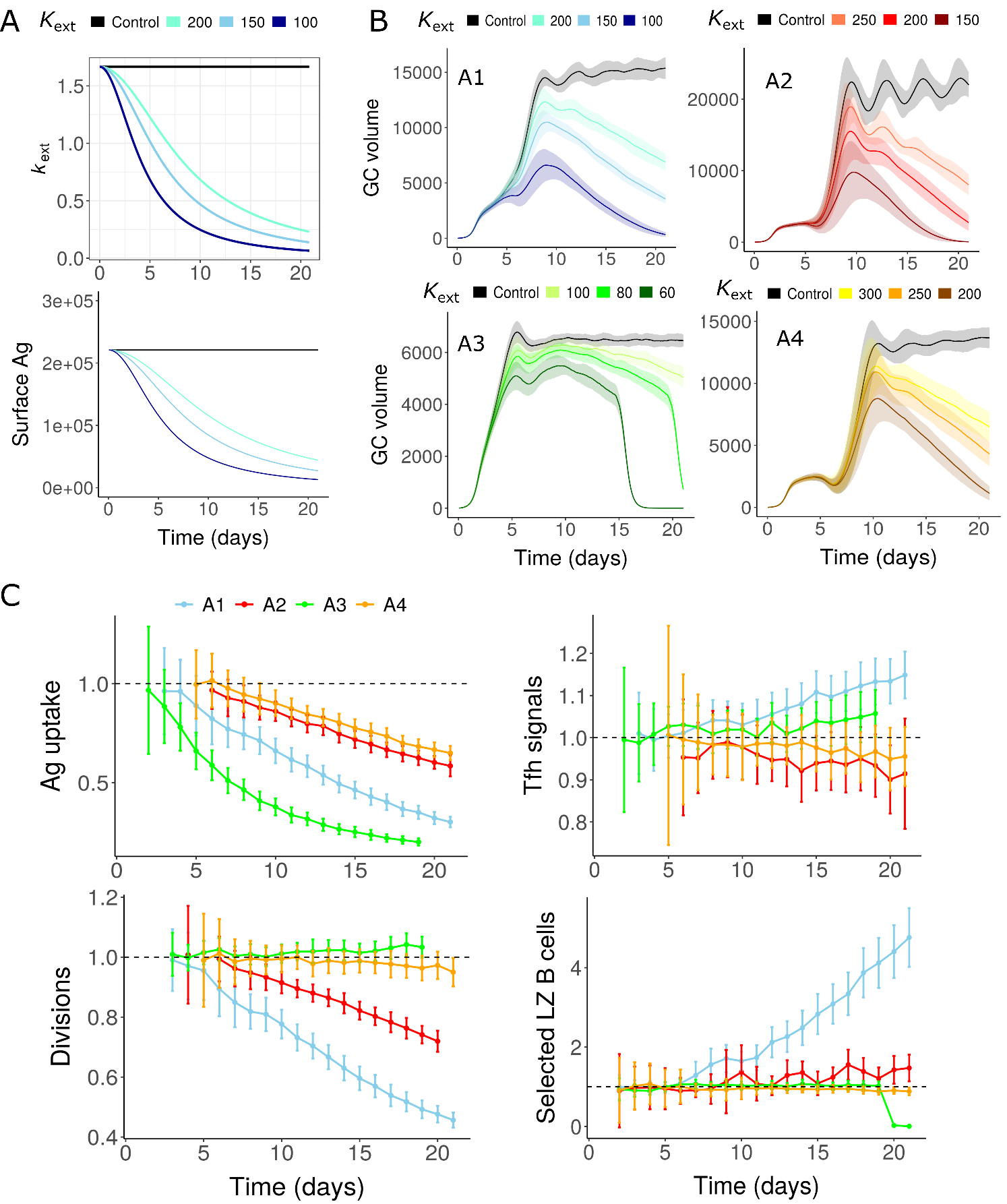


**Figure S4: GC shutdown due to modulation of antigen cycling rate constants (Mechanism 4).** A) Externalization rate constant as a function of time and surface antigen amount for assumption A1. B) GC dynamics under assumptions A1-A4. Black curves represent control simulations. Different colors represent the value of *K*_ext_ in Equation 7 as shown in the labels above panel B. $K_{\mathrm{ext}}$ is the time point in hours where the externalization rate constant of antigen decreases to half of its initial value (see Methods). In panels A and B, solid lines and shaded regions represent mean and standard deviation of 50 simulations, respectively. C) Average antigen uptake per B cell, Tfh signals acquired per B cell, number of divisions of recycling GC B cells and fraction of Tfh selected LZ B cells. Readouts were normalized with that of the control simulation. Colors represent the assumption considered (A1-A4). In panel C, values of *K*_ext_ were 150, 200, 80 and 250 in assumptions A1-A4, respectively. Error bars represent standard deviation of 50 simulations. GC: Germinal center; FDC: Follicular dendritic cell; Tfh: T follicular helper cell; LZ: Light Zone; Ag: Antigen.


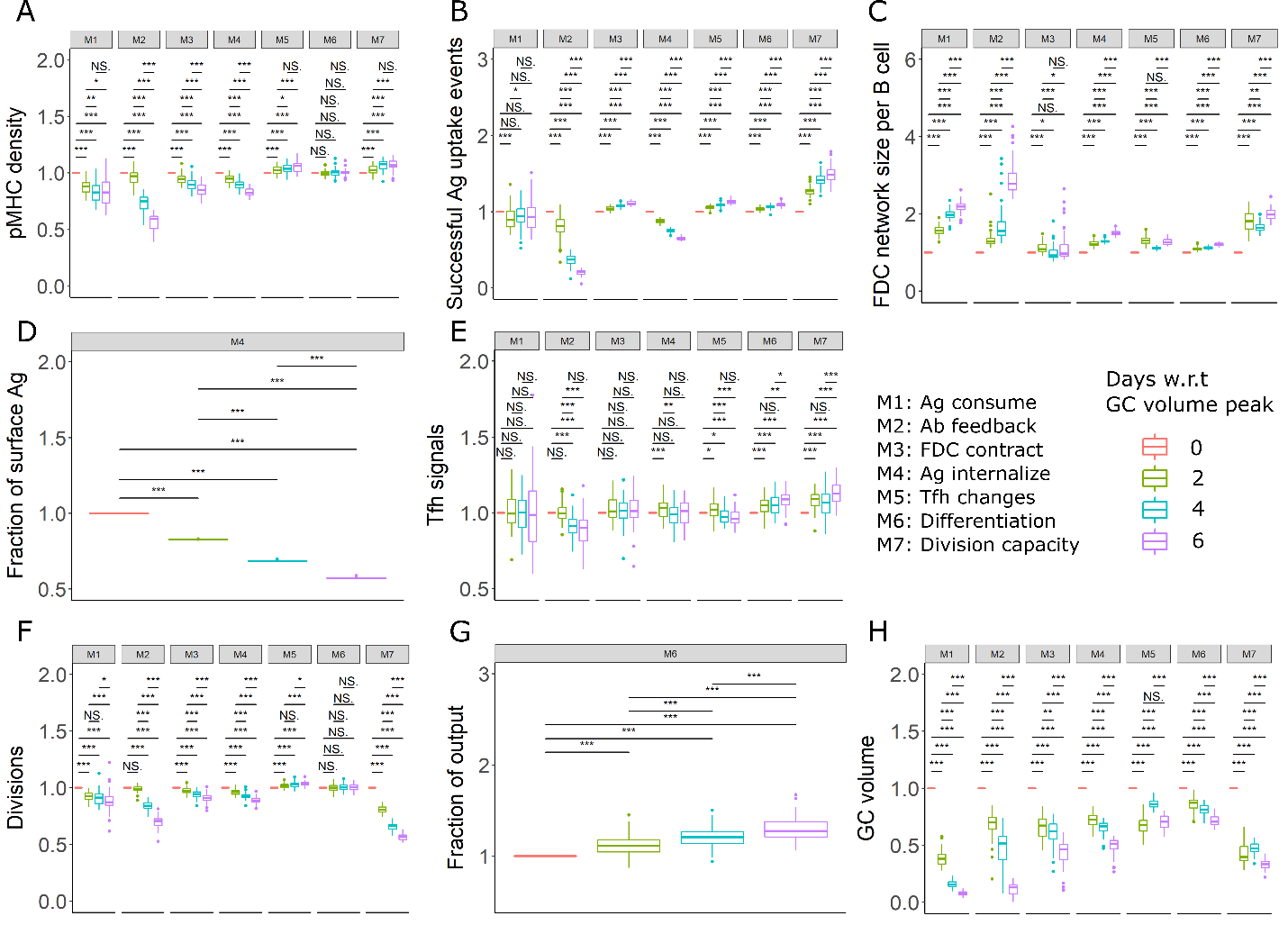


**Figure S5: In-silico analysis of differences among the mechanisms proposed with assumption A2.** A) pMHC density of selected centrocytes, B) Fraction of successful antigen uptake events among all FDC-B cell encounters, C) FDC network size per B cell, D) Fraction of Ag on FDC surface, E) Tfh signals received by selected centrocytes, F) Average number of divisions of recycling centrocytes, G) Fraction of output cells among Tfh selected cells, H) GC volume in all mechanisms. All readouts were normalized with respect to (w.r.t) the value at the peak of the GC reaction. Different colors represent the different time points with respect to GC reaction peak. The FDC network size per B cell was calculated by dividing the total number of lattice sites occupied by FDCs by the total number of GC B cells. Statistical significance was evaluated using Wilcoxon test. Error bars represent standard deviation of 50 simulations. Parameter values used in different mechanism: M1: 1 unit of Ag consumption per FDC-B cell interaction, M2: *N*=300, M3: FDC contraction rate = 0.166 µm per hour, M4: *K*_ext_ = 200, M5: *K*_T_ = 600, M6: *k* = 0.003, M7: *K*_K_ = 5. GC: Germinal center; Tfh: T follicular helper cell; Ag: Antigen; Ab: Antibody; FDC: Follicular dendritic cell. * = p < 0.05, ** = p < 0.01, *** = p < 0.001.


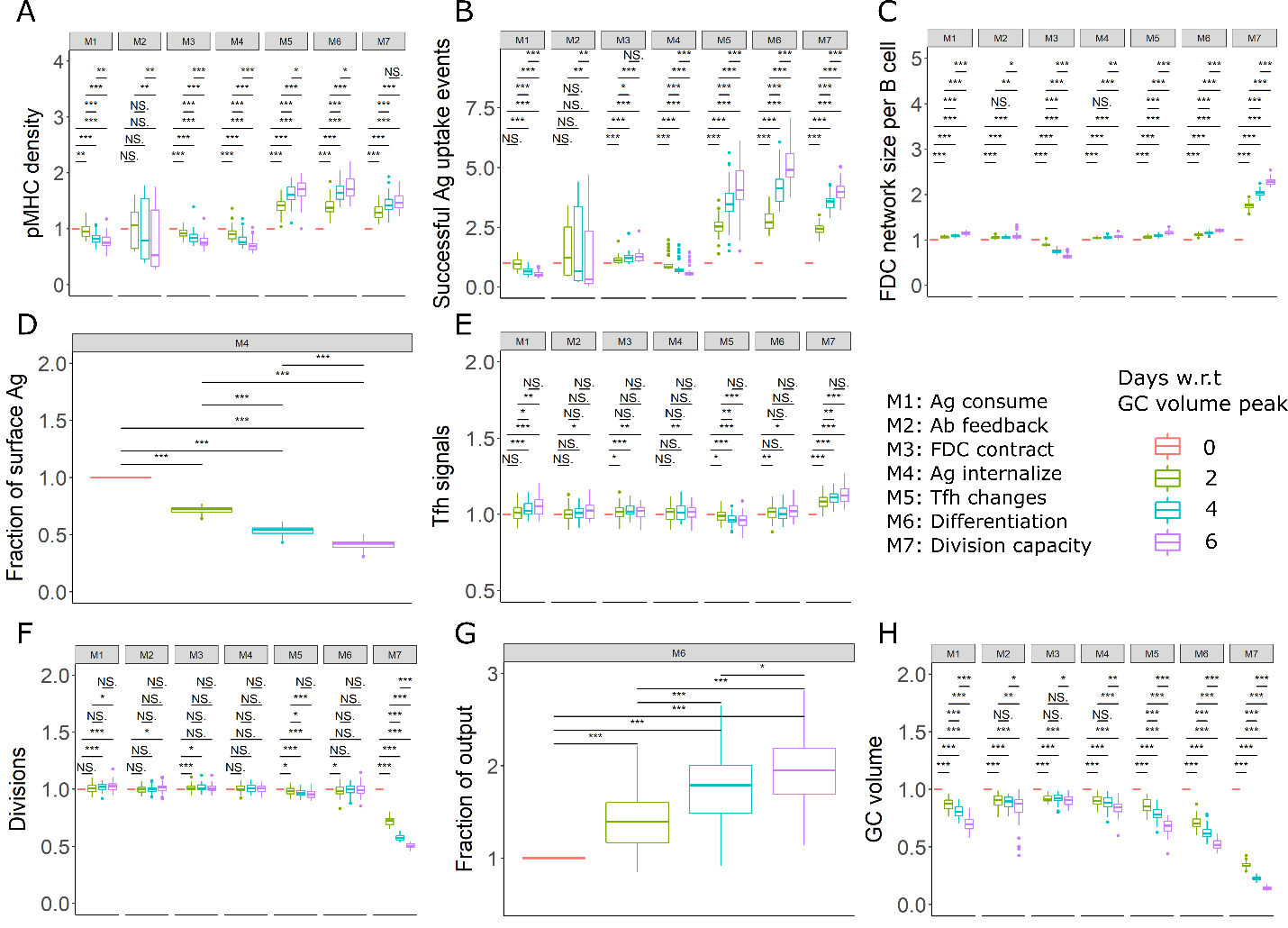


**Figure S6: In-silico analysis of differences among the mechanisms proposed with assumption A3.** A) pMHC density of selected centrocytes, B) Fraction of successful antigen uptake events among all FDC-B cell encounters, C) FDC network size per B cell, D) Fraction of Ag on FDC surface, E) Tfh signals received by selected centrocytes, F) Average number of divisions of recycling centrocytes, G) Fraction of output cells among Tfh selected cells, H) GC volume in all mechanisms. All readouts were normalized with respect to (w.r.t) the value at the peak of the GC reaction. Different colors represent the different time points with respect to GC reaction peak (see legend). The FDC network size per B cell was calculated by dividing the total number of lattice sites occupied by FDCs by the total number of GC B cells. Statistical significance was evaluated using Wilcoxon test. Error bars represent standard deviation of 50 simulations. Parameter values used in different mechanism: M1: 1 unit of Ag consumption per FDC-B cell interaction, M2: *N*=300, M3: FDC contraction rate = 0.166 µm per hour, M4: *K*_ext_ = 80, M5: *K*_T_ = 600, M6: *k* = 0.003, M7: *K*_K_ = 5. GC: Germinal center; Tfh: T follicular helper cell; Ag: Antigen; Ab: Antibody; FDC: Follicular dendritic cell. * = p < 0.05, ** = p < 0.01, *** = p < 0.001.


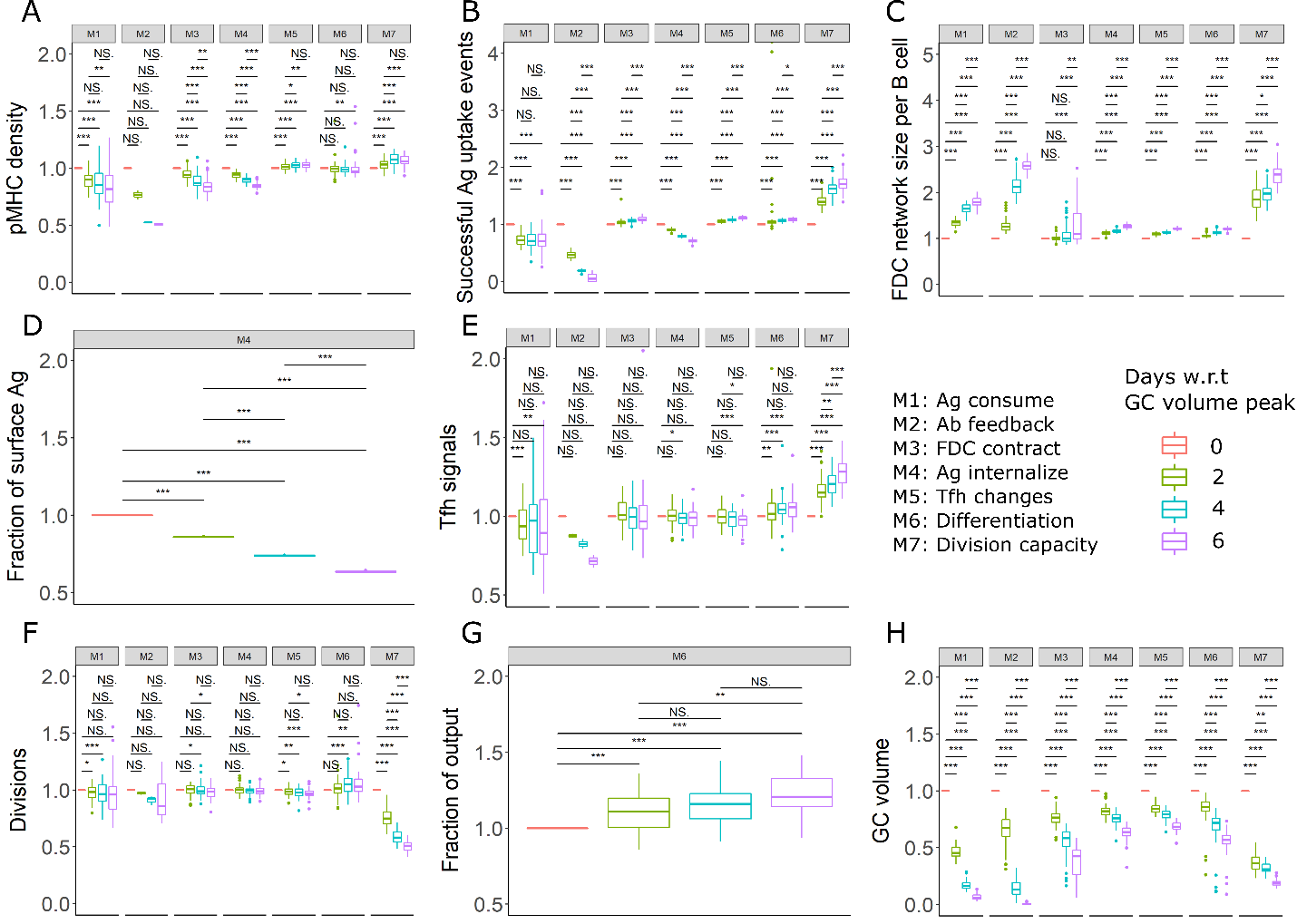


**Figure S7: In-silico analysis of differences among the mechanisms proposed with assumption A4.** A) pMHC density of selected centrocytes, B) Fraction of successful antigen uptake events among all FDC-B cell encounters, C) FDC network size per B cell, D) Fraction of Ag on FDC surface, E) Tfh signals received by selected centrocytes, F) Average number of divisions of recycling centrocytes, G) Fraction of output cells among Tfh selected cells, H) GC volume in all mechanisms. All readouts were normalized with respect to (w.r.t) the value at the peak of the GC reaction. Different colors represent the different time points with respect to GC reaction peak. The FDC network size per B cell was calculated by dividing the total number of lattice sites occupied by FDCs by the total number of GC B cells. Statistical significance was evaluated using Wilcoxon test. Error bars represent standard deviation of 50 simulations. Parameter values used in different mechanism: M1: 1 unit of Ag consumption per FDC-B cell interaction, M2: *N*=300, M3: FDC contraction rate = 0.166 µm per hour, M4: *K*_ext_ = 250, M5: *K*_T_ = 600, M6: *k* = 0.003, M7: *K*_K_ = 5. GC: Germinal center; Tfh: T follicular helper cell; Ag: Antigen; Ab: Antibody; FDC: Follicular dendritic cell. * = p < 0.05, ** = p < 0.01, *** = p < 0.001.


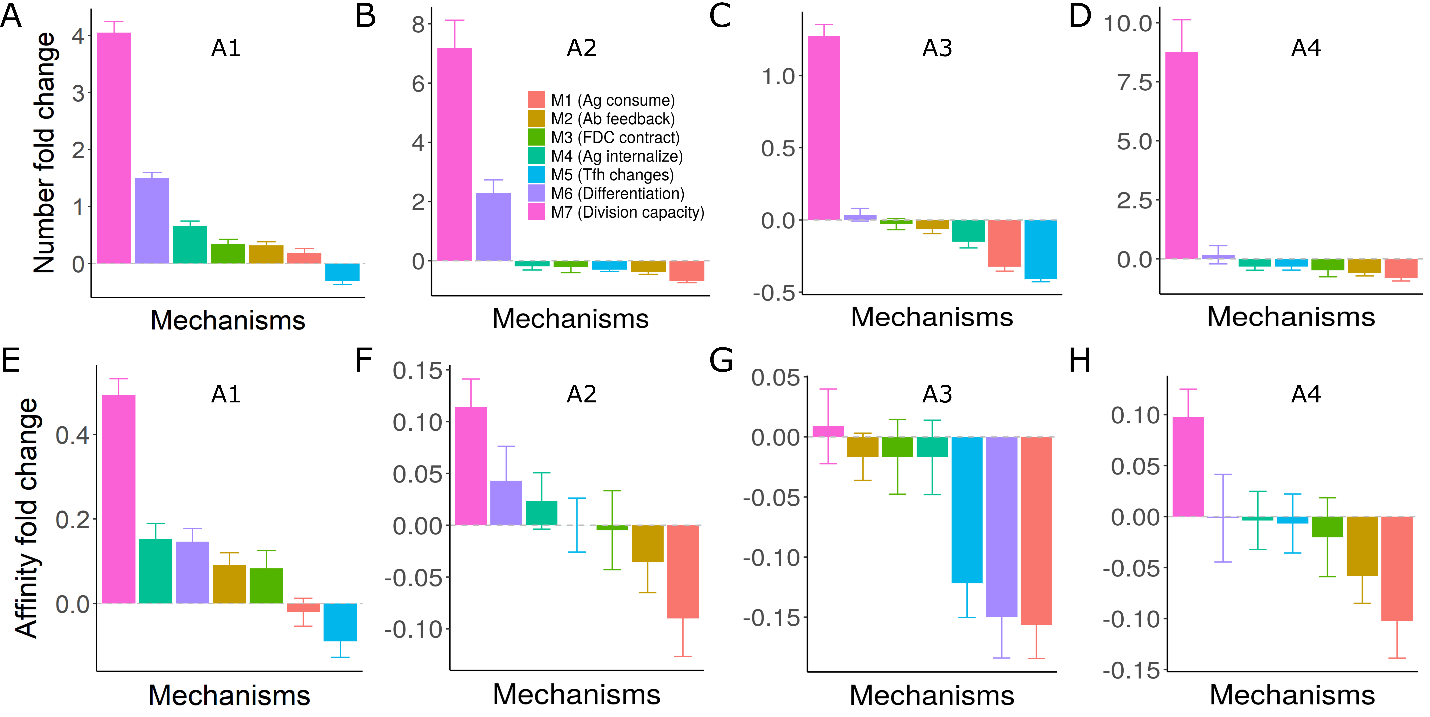


**Figure S8: Implication of GC shutdown on quality and quantity of GC output.** A-D) Fold change in number of output cells produced, and E-H) affinity of output cells in different mechanisms (colors) and for assumptions A1-A4, respectively. Fold change was calculated at day 21 of GC reaction with respect to control simulations. Error bars represent standard deviation of 50 simulations. Positive and negative values represent an increase and decrease, respectively, compared to the control simulation. Parameter values used in different mechanism: M1: 1 unit of Ag consumption per FDC-B cell interaction, M2: *N*=300, M3: FDC contraction rate = 0.166 µm per hour, M4: *K*_ext_ =150, 200, 80 and 250 in assumptions A1-A4, M5: *K*_T_ = 600, M6: *k* = 0.003, M7: *K*_K_ = 5. GC: Germinal center; Ab: Antibody; Ag: Antigen; Tfh: T follicular helper cell; FDC: Follicular dendritic cell.
